# Quantum Contextual Hypergraphs, Operators, Inequalities, and Applications in Higher Dimensions

**DOI:** 10.3390/e27010054

**Published:** 2025-01-09

**Authors:** Mladen Pavičić

**Affiliations:** 1 Center of Excellence for Advanced Materials and Sensors, Research Unit Photonics and Quantum Optics, Institute Ruder Bošković, 10000 Zagreb, Croatia; mpavicic@irb.hr; 2 Institute of Physics, 10000 Zagreb, Croatia; 3 Nano Optics, Department of Physics, Humboldt University, 12489 Berlin, Germany

**Keywords:** quantum contextuality, hypergraph contextuality, MMP hypergraphs, operator contextuality, random generation, Kochen–Specker sets, non-Kochen–Specker contextual sets

## Abstract

Quantum contextuality plays a significant role in supporting quantum computation and quantum information theory. The key tools for this are the Kochen–Specker and non-Kochen–Specker contextual sets. Traditionally, their representation has been predominantly operator-based, mainly focusing on specific constructs in dimensions ranging from three to eight. However, nearly all of these constructs can be represented as low-dimensional hypergraphs. This study demonstrates how to generate contextual hypergraphs in any dimension using various methods, particularly those that do not scale in complexity with increasing dimensions. Furthermore, we introduce innovative examples of hypergraphs extending to dimension 32. Our methodology reveals the intricate structural properties of hypergraphs, enabling precise quantifications of contextuality. Additionally, we investigate several promising applications of hypergraphs in quantum communication and quantum computation, paving the way for future breakthroughs in the field.

## 1. Introduction

Quantum contextuality is a property of a set of quantum states that precludes assignments of predetermined 0–1 values to them, i.e., it assumes the total absence of two-valued states on them. The set can be a Kochen–Specker one [1,2,3] or a non-Kochen–Specker one [4]. We will provide relevant definitions and references later on.

Contextual sets have been implemented in various experiments [5,6,7,8,9,10,11,12,13,14,15,16,17,18,19,20,21,22,23,24,25], which we will not discuss here, because most of them simply confirm that implemented contextual sets are contextual.

Quantum contextual sets have found application in quantum communication [26,27,28], quantum computation [29,30], quantum nonlocality [31], quantum steering [32], and lattice theory [33], but mainly with smallest sets and with little elaboration regarding how to scale them up. We shall discuss several application proposals and possible scale-ups later on.

First of all, we shall provide hypergraph formulations for Kochen–Specker as well as for non-Kochen–Specker contextual sets. These formulations may arise, e.g., from

- operator-based sets [34,35,36,37,38,39];
- sets built by multiples of mutually orthogonal vectors, where at least one of the multiples contains less than *n* vectors, with *n* being the dimension of space in which the sets reside [29]; or
- the so-called true-implies-false and true-implies-true sets [40,41], etc.

To make this introduction self-reliant, we need to introduce several definitions. We do not assume that the reader is familiar with the language and formalism of general hypergraphs or the particular types of hypergraphs that we employ in this paper. Therefore, we shall briefly and informally review some properties of general hypergraphs, followed by a more rigorous definition of the specific types that we use.

### 1.1. General Hypergraphs

A general hypergraph is a pair of a finite set of elements and a family of subsets of these elements. The elements are called vertices of the hypergraph, and the subsets are called hyperedges of the hypergraph. Vertices might be represented by vectors, operators, numbers, geometrical points, files in a database, elements of a DNA sequence, or other objects, and hyperedges are represented by a relation between the vertices contained in them, such as orthogonality, inclusion, geometry, data records, genes, etc. [42,43,44,45]. Thus, a set of *vertices*, $V=\{v_{1},v_{2},\ldots ,v_{k}\}$ and a set of subsets of *V* (called *hyperedges*: $e_{i}$, i=1,…,l), $E=\{e_{1},e_{2},\ldots ,e_{l}\}$, build a pair H=(V,E) called a *hypergraph*; notations V(H) and E(H) are also in use. Graphically, a hyperedge $e_{j}$ may be represented as a continuous curve joining two elements/points/vertices if the cardinality (number of elements) within the hyperedge is $|e_{j}|=2$, by a loop if $|e_{j}|=1$, and by a closed curve enclosing the elements if $|e_{j}|>2$. Numerically, they can be represented by the incidence matrices ([43], p. 2, Figure 1), with columns as hyperedges and rows as vertices. Intersections of hyperedge columns with vertex rows contained in hyperedges are assigned 1, and those not contained are assigned 0.

The number of vertices within a hypergraph (*k*), i.e., the cardinality of *V* (|V|), is called the *order* of a hypergraph, and the number of hyperedges within a hypergraph (*l*), i.e., the cardinality of *E* (|E|), is called the *size* of a hypergraph.

### 1.2. *MMP* Hypergraphs

The particular type of general hypergraph that we deal with in this paper is the McKay–Megill–Pavičić hypergraph (MMPH).

**Definition 1.**  **MMPH-dimension** *n (*MMPH-dim* n) is a predefined (for an assumed task or purpose) maximal possible number (n) of vertices within a hyperedge of an* MMPH*, even when none of the processed hyperedges include n vertices. It is abbreviated as* MMPH-dim.

**Definition 2.**  *An* **MMPH** *is a connected hypergraph H=(V,E) (where $V=\{V_{1},V_{2},\ldots ,V_{k}\}$ is a set of *vertices *and $E=\{E_{1},E_{2},\ldots ,E_{l}\}$ sets of* hyperedges*) of* MMPH-dim* n≥3 in which*

*1.*  *Every vertex belongs to at least one hyperedge;*
*2.*  *Every hyperedge contains at least 2 and at most n vertices;*
*3.*  *No hyperedge shares only one vertex with another hyperedge;*
*4.*  *Hyperedges may intersect with each other in at most n−2 vertices;*
*5.*  *Numerically, an* MMPH* is a string of ASCII characters corresponding to vertices and organized in substrings separated by commas (“,”), corresponding to hyperedges; the string ends with a period (“.”); one uses 90 characters:* 1...9 A...Z a...z!"#$%&’( ) * - / : ; < = > ? @ [ ∖ ] ^ _ ‘ { | } ∼*; when exhausted, one reuses them prefixed by ‘+’ and then by ‘++’, etc.; there is no limit on their length;*
*6.*  *Graphically, vertices are represented as dots and hyperedges as (curved) lines passing through them.*

**Figure 1:**
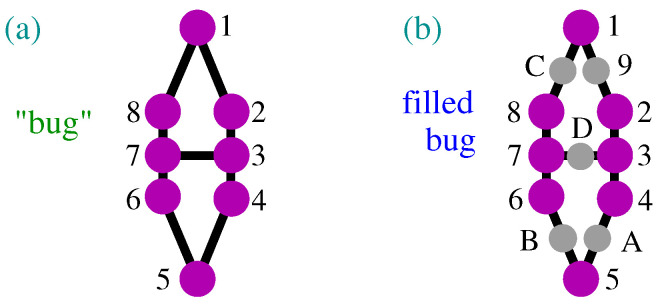
(**a**) The 8-7 NBMMPH ([50], Supplementary Materials, Figure 3) or the *bug*; cf. ([51], $\Gamma_{1}$); (**b**) filled bug—13-7 BMMPH; grey dots represent vertices with m=1.

The differences between the standard hypergraph formalism and the MMPH formalism are illustrated in ([3], Figure 1).

### 1.3. Non-Binary and Binary *MMPH*s

**Definition 3.**  *A k-l* MMPH *of dim n≥3 with k vertices and l hyperedges, whose j-th hyperedge contains κ(j) vertices (2≤κ(j)≤n, j=1,…,l), to which it is impossible to assign 1s and 0s in such a way that the following* rules *hold*

*(i)*  *No two vertices within any of its hyperedges may both be assigned the value* 1*;*
*(ii)*  *In any of its hyperedges, not all vertices may be assigned the value* 0*.*

*is called a* **non-binary** MMPH* (***NBMMPH***).*

An NBMMPH is contextual as it does not allow predetermined values 1 and 0 to be assigned to all vertices by violating rules (*i*) and (*ii*).

**Definition 4.**  *An* MMPH *to which it is possible to assign 1s and 0s to satisfy rules (i) and (ii) of Definition 3 is called a* **binary** MMPH *(***BMMPH***).*

A BMMPH is noncontextual as it does allow predetermined values 1 and 0 to be assigned to all vertices by satisfying rules (*i*) and (*ii*).

**Definition 5.**  *A* **critical NBMMPH** *is an* NBMMPH *that is minimal in the sense that removing any of its hyperedges transforms it into a* BMMPH.

**Definition 6.**  **Vertex multiplicity** *is the number of hyperedges that vertex ‘i’ belongs to; we denote it by m(i).*

**Definition 7.**  *A* **master** *is a non-critical* MMPH *that contains smaller critical and non-critical sub-*MMPH*s. A collection of all sub-*MMPH*s of an *MMPH *master forms its* class.

**Definition 8.**  *Let K be a subset of an* MMPH *H=(V,E). A K from which at least one of vertices with vertex multiplicity m=1 is taken out is called a $\bar{\mathrm{subhypergraph}}$.*

### 1.4. Hypergraph Structural Discriminators

The principal distinction between an NBMMPH and a BMMPH is that, in the latter, we can assign 1s to vertices to satisfy conditions *(i)* and *(ii)* from Definition 3 and that all hyperedges contain one of them, while, in the former, this is not possible since there exists at least one hyperedge where the conditions are violated, preventing any vertex from being assigned the value 1. It has been found that the number of 1s that one can assign to vertices in an NBMMPH vs. a BMMPH plays a crucial role in discriminating between these two MMPHs and in evaluating the contextuality of the former.

**Definition 9.**  *The* **MMPH classical vertex index ***$HI_{c}$ is the number of* 1*s that one can assign to the vertices of an *MMPH* so as to satisfy conditions (i) and (ii) from Definition *3*. The maximal (minimal) $HI_{c}$ is denoted as $HI_{cM}$ ($HI_{cm}$).*

(Note: In ref. [46], some values of $HI_{c}$ were incorrectly calculated due to an application problem in our previous algorithm and program; algorithm and program One used since (see [3,47]) are substitutes for the previous ones).

**Definition 10.**  *The* **MMPH classical multiplexed vertex index** *$HI_{c}^{m}$ is the number that we obtain when summing up all multiplicities of vertices of an n-dim* MMPH*, whose every hyperedge contains n vertices, to which we can assign 1s so as to satisfy conditions (i) and (ii) from Definition 3. The maximal (minimal) $HI_{c}^{m}$ is denoted as $HI_{cM}^{m}$ ($HI_{cm}^{m}$).*

We obtain $HI_{c}$ and $HI_{c}^{m}$ through the algorithm and its program One, which assigns 1s to the vertices of an MMPH. The algorithm randomly searches for a distribution of 1s satisfying conditions *(i)* and *(ii)* from Definition 3. It starts with a randomly chosen hyperedge in which one vertex is assigned 1 and the other vertices are assigned 0s and continues with the connected hyperedges until all permitted vertices are assigned 1. Multiplicities for the found 1s accumulated in the process are taken into account. For NBMMPHs, this means “until a contradiction is reached”, i.e., to a point at which no vertex from the remaining hyperedges can be assigned 1; vertices within the latter hyperedges are all assigned 0s. The maximal number of 1s ($HI_{cM}$, $HI_{cM}^{m}$) is obtained by (up to 50,000) parallel runs with reshuffled vertices and hyperedges. Because we do not make use of backtracking in the search algorithm to resolve conflicts, the procedure does not exponentially increase the CPU time with an increasing number of vertices. MMPHs with several thousand vertices and hyperedges are processed within seconds on each CPU of a cluster or a supercomputer.

The probability of not finding the correct minimal or maximal $HI_{c}$ and $HI_{c}^{m}$ after so many runs is extremely small; nevertheless, this small probability restrains our results, meaning that slightly larger maxima and smaller minima might be found in future computations for a chosen hypergraph.

**Definition 11.**  *The* **classical hyperedge number $l_{c}$** *is the number of hyperedges that contain vertices that take part in building $HI_{c}$, and the maximal and minimal number of such hyperedges are $l_{cM}$ and $l_{cm}$, respectively.*

We stress that, in most cases, $l_{cM}$ hyperedges do not contain $HI_{cM}$ vertices but a smaller number of them. Moreover, $l_{cm}$ hyperedges usually do not contain $HI_{cm}$ vertices but a larger number of them.

The classical vertex index $HI_{cM}$ of a hypergraph H is related to the independence number of H introduced by Grötschel, Lovász, and Schrijver (GLS) ([48], p. 192). They introduced the definition for graphs, but it holds for hypergraphs as well, with graph cliques transliterated into hyperedges.

**Definition 12.**  **GLS α** *. The independence number of H denoted by α(H) is the maximum number of pairwise non-adjacent vertices.*

The independence number α has been given several definitions and names in the literature. For instance, “α(H) is the size of the largest set of vertices of H such that no two elements of the set are adjacent” [29]. Such a set is called an *independent* or a *stable* set ([49], Definition 2.13) ([42], pp. 272, 428), and α is also called a *stability number* ([42], pp. 272, 428). In such a set, no two vertices are connected by a hyperedge. The definitions of these notions given by Voloshin differ since his sets might include two or more vertices from the same hyperedge ([45], p. 151).

**Lemma 1.**  *$HI_{cM}\left(H\right)=\alpha \left(H\right)$.*

**Proof.**  Via conditions (i) and (ii) from Definition 3 which Definition 9 invokes, no two vertices to which one can assign 1 can belong to the same hyperedge. The maximum number of such vertices, i.e., $H_{cM}\left(H\right)$, is therefore the maximum number of pairwise non-adjacent vertices, i.e., according to Definition 12, just α(H).   □

### 1.5. Coordinatization

Vector or state or operator representation, i.e., a *coordinatization* of vertices, is operationally required for any implementation of an MMPH since a full coordinatization of vertices transforms MMPH-dim *n* into a dimension of a Hilbert space determined by the vectors that each vertex is assigned to. Whether we refer to an MMPH with or without a coordinatization will be clear from the context.

An *n*-dim *k*-*l* NBMMPH H need not have a coordinatization, but, when it does, the vertices in every hyperedge have definite mutually orthogonal vectors assigned to them. This means that each hyperedge $E_{j}$, j=1,…,l should have not only κ(j) vectors corresponding to its κ(j) vertices specified, but also n−κ(j) ones that must exist in each $E_{j}$ by the virtue of orthogonality in the *n*-dim space, so as to form an orthogonal basis of the space. Such an extended H is called a *filled* H. We can have a filled MMPH even when a coordinatization does not exist. Then, it means that, to each hyperedge that does not contain *n* vertices, vertices are added so that it does.

**Definition 13.**  *A* **filled** *n-dim* MMPH *is one that is derived from an n-dim* MMPH *with at least one hyperedge containing fewer than n vertices by adding vertices to ensure that all hyperedges have precisely n vertices.*

For instance, a contextual critical NBMMPH 12,234,45,56,678,81,37, or a “bug”, as shown in Figure 1a, obtains a coordinatization from its filled MMPH 192,234,4A5,5B6,678, 8C1,3D7. The latter MMPH is not contextual; it is a BMMPH, as shown in Figure 1b.

Our algorithms and programs can detect the contextuality of an MMPH regardless of whether its coordinatization is given (or even existent) or not. The handling of MMPHs using our algorithms embedded in the programs SHORTD, MMPSTRIP, MMPSUBGRAPH, VECFIND, STATES01, and others, without taking their coordinatization into account, gives us a computational advantage over handling them with a coordinatization, because processing bare hypergraphs is faster than processing them with vectors assigned to their vertices.

**Definition 14.**  *A* coordinatization *of an* MMPH *is a set of vectors/states assigned to its vertices, which is a subset of n-dim vectors in $H^{n}$, n≥3, assigned to its vertices, provided that all hyperedges contain n vertices, or to vertices of its filled *MMPH *or of any of its *MMPH *masters whose hyperedges all contain n vertices.*

Hence, an NBMMPH whose hyperedges contain m≤n vertices inherits its coordinatization from the coordinatization of its master or of its filled set (they may coincide, but usually they do not). When the method **M1** (from Section 2.2) for the generation of MMPHs is applied, a coordinatization is automatically assigned to each contained MMPH by the very procedure of its generation (method **M2**) from master MMPHs, as we shall see below.

Note that the Kochen–Specker theorem below does not require a coordinatization, although its original proof involved one [51].

**Theorem 1.**  **Kochen–Specker Theorem.** *There exist k-l *MMPH*s of dim n≥3 with k vertices and l hyperedges, whose j-th hyperedge contains κ(i) vertices (2≤κ(j)≤n, j=1,…,l) to which it is impossible to assign 1s and 0s in such a way that the following* rules *hold:*

*(i)*  *No two vertices within any of its hyperedges may both be assigned the value* 1*;*
*(ii)*  *In any of its hyperedges, not all vertices may be assigned the value* 0*.*

*Such* MMPH*s are called*** KS MMPH***s. All* KS MMPH*s are* NBMMPH*s. Every* KS MMPH* is a* **proof** *of the* KS* theorem.*

**Proof.**  Obvious [1,3,51,52].   □

This paper is organized as follows.

In Section 2.1, we introduce Kochen–Specker (KS) and non-Kochen–Specker (non-KS) NBMMPHs.

In Section 2.2, we present eight methods, **M1–8**, which we make use of to generate MMPHs.

In Section 2.3, we consider the operator approach, which yields several types of inequalities, and we prove several lemmas on them. We also consider the fractional independence (packing) number $\alpha^{*}\left(H\right)$ of an MMP and arrive at the Quantum Indeterminacy Postulate 1 and two different types of statistics, Definitions 21 and 22, which satisfy Theorem 2.

In Section 2.4, we review special cases of KS and non-KS MMPHs in dimensions 3 to 32 and provide new instances of them. We also introduce a new graphical presentation of higher-dimensional MMPHs.

In Section 2.5, we give four possible applications of higher-dimensional MMPHs. Section 2.5.1 considers larger alphabet communication, and we provide a detailed protocol for it; Section 2.5.2 presents the oblivious communication protocol; Section 2.5.3 discusses generalized Hadamard matrices; and Section 2.5.4 discusses stabilizer operations.

In Section 3, we discuss the results obtained in this article.

## 2. Results

We conduct an in-depth analysis of contextual sets, focusing on their structure and properties. Our aim is not to create blueprints for experiments that prove their contextuality (because, after so many experiments carried out so far, new ones would not reveal anything new) or to use contextuality do disprove hidden variable models (which are now barely regarded as relevant). We also do not seek to derive new inequalities, as existing tools already allow us to demonstrate contextual sets efficiently. Lastly, we do not aim to design BB84-like cryptographic contextual protocols because they cannot provide a quantum advantage over the noncontextual protocols; an eavesdropper can easily ignore conditions *(i)* and *(ii)* of Definition 3 and mimic quantum measurement outcomes.

Instead, we are interested in the structure and properties of contextual sets and their generation. As for the generation of the sets we focus on several methods in dimensions up to eight and on the methods whose complexity does not scale with dimension to obtain sets in higher dimensions (here up to 32) by using sets generated in lower dimensions by the previous methods. While carrying out such a unification of methods, we also discuss the realistic implementation of the sets themselves and their applications, as well as the tools used to manipulate them.

### 2.1. Kochen–Specker vs. Non-Kochen–Specker *MMPH*s

When considering implementations or applications of MMPHs, we primarily focus on a set of quantum states represented by vectors in *n*-dim Hilbert space, organized into *m*-tuples (where m≤n) of mutually orthogonal vectors, with at least one hyperedge containing m=n vertices. We impose the latter requirement because we believe that it is plausible to assume that the considered vertices (vectors, states, operators) fully reside in the *n*-dim space within at least one of the hyperedges, although there are several exceptions to this requirement in the literature, the most notable one being the 2-dim pentagon in the 3-dim space ([3], Figure 5, [34]). For the same reason, we will not try to equip *n*-dim MMPHs without a coordinatization with a coordinatization from a higher-dimensional space, although examples of such an embedding do exist in the literature too ([3], Figure 4, [53]).

**Definition 15.**  *An n-dim* NBMMPH *with a coordinatization, in which each hyperedge contains m = n vertices, is a Kochen–Specker* (KS) MMPH.

**Definition 16.**  *An n-dim *NBMMPH* with a coordinatization, in which at least one hyperedge contains m<n vertices and at least one hyperedge contains m = n vertices, is a *non-KS MMPH.

Both KS and non-KS MMPHs are NBMMPHs and are therefore contextual.

### 2.2. Generation of *NBMMPHs*

To generate NBMMPHs, we make use of the following methods.

- **M1**: Combines simple vector components to exhaust all possible collections of *n* mutually orthogonal *n*-dim vectors. These vectors form *master* MMPHs, which consist of single or multiple MMPHs of varying sizes. Master MMPHs may or may not be NBMMPHs [50,54].
- **M2**: Automated dropping of hyperedges contained in masters found by **M1, M6–8** or by some other method in the literature; they serve to generate *classes* of smaller MMPHs massively [50,54].
- **M3**: Automated dropping of vertices contained in single hyperedges (multiplicity m=1) of either NBMMPHs or BMMPHs and the possible subsequent stripping of their hyperedges [3]. The obtained smaller MMPHs are often NBMMPH, although never KS.
- **M4**: Automated random addition of hyperedges to MMPHs to obtain larger ones, which then generates smaller KS MMPHs through the random removal of hyperedges again.
- **M5**: Deleting vertices in either an NBMMPH or a BMMPH until a non-KS NBMMPH is reached, if any.
- **M6**: Downward generation of NBMMPHs from fortuitously or intuitively found connections of KS MMPHs with polytopes, Pauli operators, or space symmetries [3,55,56].
- **M7**: Generation of KS MMPHs in higher dimensions from the ones in smaller dimensions [1,2,57,58].
- **M8**: Generation of KS MMPHs in higher dimensions by dimensional upscaling, whose complexity does not scale with the dimension [47].

We combine all of these methods to obtain an arbitrary number of NBMMPHs in an arbitrary dimension and to generate KS and non-KS MMPHs in dimensions up to 32 below.

**Figure 2:**
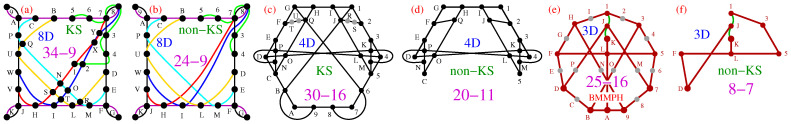
Obtaining non-KS MMPHs via three different methods (colored lines represent hyperedges): (**a**) 8-dim KS MMPH obtained via **M1** or **M6**; (**b**) 8-dim non-KS MMPH $\bar{\mathrm{subhypergraph}}$ of (**a**) obtained by **M5** (deleting vertices inside the rectangle loop); (**c**) 4-dim KS MMPH obtained via **M1** or **M6**; (**d**) 4-dim non-KS MMPH $\bar{\mathrm{subhypergraph}}$ obtained from (**c**) by **M2** and **M3** (successive deletions of m=1 (grey) vertices and hyperedges so that, at each step, the resulting MMPH is a non-KS MMPH—until we reach the smallest critical non-KS without m=1 vertices); (**e**) 3-dim (noncontextual) BMMPH subhypergraph obtained by **M1** and **M2**; (**f**) 3-dim non-KS MMPH $\bar{\mathrm{subhypergraph}}$ obtained from (**e**) by **M2** and **M3** (successive deletions of vertices and hyperedges so that, at each step, the resulting MMPH is a non-KS MMPH—until we reach a critical non-KS without m=1 vertices); strings and coordinatizations of (**a**,**c**,**e**) are given in the Appendix A since they were not given elsewhere; strings and coordinatizations of (**b**,**d**,**f**) can be derived from those of (**a**,**c**,**e**).

To familiarize ourselves with non-KS MMPHs, in Figure 2, we visualize the procedures of obtaining them from two types of KS MMPHs and (noncontextual) BMMPHs by means of our methods.

The 8-dim KS MMPH shown in Figure 2a is obtained via **M6** from the 120–2025 master but can also be obtained from the 3280–1361376 master directly generated from the {0,±1} vector components, i.e., via **M1** [54]. The 4-dim KS MMPH, in Figure 2c, is obtained via **M6** from the 60–105 Pauli operator master [55], but can also be obtained from the 156–249 master directly generated from the {0,±1,±i} vector components, i.e., via **M1** [54]. The 3-dim noncontextual BMMPH, in Figure 2e, is obtained via **M6** from the Peres master, but can also be obtained from the 81–52 master directly generated from the $\left\{0,\pm 1,\sqrt{2},\pm 3\right\}$ vector components, i.e., via **M1** [50].

One can easily verify by hand that the MMPHs shown in Figure 2b,d,f violate the conditions *(i)* and *(ii)* in Definition 3, i.e., that they are NBMMPHs and that they, by having coordinatizations, are, by Definition 16, non-KS MMPHs.

### 2.3. *NBMMPHs* vs. Operators and States—The Inequalities

In the literature, contextual sets, mostly KS ones, have often been formulated by means of operators (mostly projectors) and states, especially together with a proposed implementation.

Recently, it was shown that “a proof of the Kochen–Specker theorem can always be converted to a state-independent [operator] noncontextuality inequality” [59,60]. Proofs of the KS theorem are KS sets, and the result actually means that, for any KS set with a coordinatization, i.e., for any KS MMPH with a coordinatization, we can find quantum operators whose particular expressions would give the same result regardless of the states that they are applied to. More precisely, one can form expectation values that correspond to the measurement values of these expressions and differ from the expectation values of the assumed classical counterparts. The inequality between these two expectation values, quantum and classical, is called a noncontextuality inequality.

Obviously, this result does not apply to KS sets without a coordinatization, and the question arises of whether we can form similar discriminators, i.e., some inequalities for hypergraphs without reference to coordinatization, even when the hypergraphs do possess it. To arrive at an answer to this question, we have to introduce a few definitions. But before doing so, we give an example of a state-independent operator setup of a KS set and its noncontextuality inequality.

In ref. ([61], Equation (2)), the following 4-dim operators are defined

(1) $$\begin{array}{c} A_{ij}=2|v_{ij}\rangle \langle v_{ij}|-I \end{array}$$

through the vector coordinatization of the 4-dim KS 18-9 MMPH shown in ([61], Figure 1), e.g.,

(2) $$\begin{array}{c} |v_{12}\rangle =\left(1,0,0,0\right);\;\;|v_{16}\rangle =\frac{1}{\sqrt{2}}\left(0,0,1,-1\right);\;\;\;\;\ldots \;\;|v_{58}\rangle =\frac{1}{\sqrt{2}}\left(1,0,-1,0\right);\;\;\ldots \end{array}$$

The reader can find all vectors in ([61], Figure 1). We do not show all vectors here because we only need one of them for our Equation (5) below. In ref. ([61], Equation (2)), it is then claimed that

(3) $$\begin{array}{c} A_{ij}A_{ik}A_{il}A_{im}=-I, \end{array}$$

where i=1,…,9 ranges over 9 hyperedges of the 18-9 KS MMPH, and j,k,l,m ranges over the vertices within each of the hyperedges, which yields (full Ω is given in ([61], Equation (1)))

(4) $$\begin{array}{c} -\langle A_{12}A_{16}A_{17}A_{18}\rangle -\cdots -\langle A_{29}A_{39}A_{59}A_{69}\rangle =\langle \Omega \rangle , \end{array}$$

would take us to a “state-independent” noncontextuality inequality. Specifically, it is argued that a quantum interpretation of $A_{ij}$ that relies on “measurements of subsets of compatible observables on different subensembles prepared in the same state” yields 〈Ω〉=9, while a classical interpretation of $A_{ij}$ (which assigns −1 or 1 to them) yields a 〈Ω〉≤7 inequality. The problem with this argumentation is that each $A_{ij}$ itself is not “state-independent”, e.g.,

(5) $$\begin{array}{c} A_{12}|v_{58}\rangle =A_{12}\frac{1}{\sqrt{2}}\left(1,0,-1,0\right)=-\frac{1}{\sqrt{2}}\left(0,0,1,0\right), \end{array}$$

i.e., $|v_{58}\rangle$ is not an eigenvector of $A_{12}$. This is important because we must have correspondence between the quantum $A_{ij}$ and classical $A_{ij}$—they both have to be measurable and the measurement outcome should be ±1 for both. This is the essence of the KS theorem.

A similar problem exists with most of the proposed state-independent operator assessments of contextual sets. They all involve products of operators that assume the passing of the states through several devices and therefore prevent them from being measured at each of them separately [3,12,35,59,62], while they are expected to yield a noncontextuality inequality. However, contextual sets actually do not require operator representation, as there is always an NBMMPH formulation for any of the sets and this formulation only requires measurements of bare states for any implementation and an algorithmic check of the data for contextuality verification.

**Figure 3:**
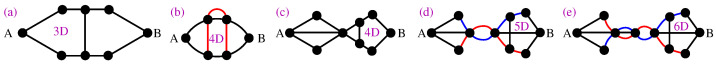
TIF diagrams according to figures from [40] (except (**b**)) (colored lines represent hyperedges): (**a**) ([40], Figure 1a), cf. Figure 1; (**b**) 4-dim NBMMPH; (**c**) ([40], Figure 4a); (**d**) ([40], Figure 5a); (**e**) ([40], Figure 7a); (**a**,**c**,**d**,**e**) are non-KS MMPHs; (**b**) would be a KS MMPH if it had a coordinatization, but it does not, so it is simply a NBMMPH.

Another approach to contextuality is provided by the so-called true-implies-false (TIF) and true-implies-true (TIT) sets [40,41], some of whose diagrams are presented in Figure 3. For instance, in TIF diagrams, if we assign value 1 (TRUE) to A and proceed to assign values so as to satisfy conditions *(i)* and *(ii)* from Definition 3 along all lines simultaneously, we find that B must be assigned value 0 (FALSE).

One can verify that all TIF and TIT diagrams from [40,41] are non-KS NBMMPHs.

To obtain a better insight into the properties of MMPHs, let us proceed.

**Definition 17.**  *The* **MMPH Quantum Hypergraph Index** *$HI_{q}$ is the sum of the weighted probabilities of all vertices of an n-dim k-l* MMPH *measured repeatedly in all hyperedges that they belong to, whenever their multiplicity is >1.*

**Lemma 2.**  **Vertex-Hyperedge Lemma.** *For any n-dim k-l* MMPH* in which each hyperedge contains n vertices, the following holds:*

(6) $$\begin{array}{c} {HI}_{q}=\sum_{i=1}^{k}\frac{m(i)}{n}=l. \end{array}$$

*In general, for any n-dim k-l* MMPH* with κ(j) considered vertices in the j-th hyperedge, j=1,…,l, the following holds:*

(7) $$\begin{array}{c} {HI}_{q}=\sum_{j=1}^{l}\sum_{\lambda =1}^{\kappa (j)}p\left(j,\lambda \right)=l, \end{array}$$

*where κ(j) is the number of vertices in a hyperedge j and $p\left(j,\lambda \right)=\frac{1}{\kappa (j)}$ is the probability that a state of a system corresponding to one of the vertices would be detected when the hyperedge j is being measured.*

**Proof.**  Equation (6) is equivalent to a generalized Handshake Lemma for Hypergraphs ([63], Exercise 11.1.3.a). The proof is given in [3].

To prove Equation (7), we simply note that $\sum_{\lambda =1}^{\kappa (j)}p\left(j,\lambda \right)=1$ for any *j*.   □

**Definition 18.**  *The* **v-inequality.** *An* MMPH* vertex inequality or simply v-inequality is defined as *

(8) $$\begin{array}{c} HI_{cm}\leq HI_{cM}\leq HI_{cM}^{m}<HI_{q}=l. \end{array}$$

**Lemma 3.**  *All n-dim* NBMMPH*s satisfy the v-inequality.*

**Proof.**  In an NBMMPH, the maximal number of hyperedges that contain ‘1’ must be smaller than the total number of hyperedges *l* by definition.   □

**Lemma 4.**  *The* **e-inequality.** *$l_{cM}$ ($l_{cm}$) satisfies the following hyper***e***dge inequality or simply* **$e_{Max}$-inequality** *(***$e_{min}$-inequality***):*

(9) $$\begin{array}{c} l_{cM}<l\;\left(l_{cm}<l\right). \end{array}$$

**Proof.**  They are noncontextuality inequalities simply because $l_{cm}=l_{cM}=l$ for all binary MMPHs by definition.   □

Apparently, $l_{cm}$ is the “rank of contextuality” [64], introduced as a quantifier of contextuality for hypergraphs. Both $e_{Max}$- and $e_{min}$-inequalities can simply be called e-inequalities.

**Lemma 5.**  *All n-dim non-binary* MMPH*s satisfy the e-inequalities.*

**Proof.**  For KS MMPHs, it follows directly from the KS theorem, since both the maximal and minimal number of hyperedges that contain 1 must be smaller than the total number of hyperedges *l*. For non-KS NBMMPHs, it follows from Definition 3 and its conditions *(i)* and *(ii)* in the same way.   □

**Definition 19.**  Fractional independence (packing) number $\alpha^{*}\left(H\right)$ (LP) *of an* MMPH* H(k-l) is the optimum value of the following linear programming problem LP=LP(H)*

*        (LP) Maximize $\sum_{v\in V}x\left(v\right)$*

*                  subject to $\sum_{v\in e}\leq 1$, ∀e∈E*

*                                    x(v)∈[0,1], ∀v∈V*

**Definition 20. Fractional independence number**  $\alpha^{*}\left(H\right)$ is defined as (Wolfram [65]):

(10) $$\begin{array}{c} \alpha^{*}\left(H\right)=\max_{\sum_{v\in e}\leq 1,\forall e\in E}\sum_{v\in V}x\left(v\right);\;x\left(v\right)\in \left[0,1\right]. \end{array}$$

If the *v*- and *e*-inequalities are satisfied, an MMPH would be contextual. If not, it would not. Thus, the *v*- and *e*-inequalities are noncontextuality inequalities.

On the other hand, there is also the following inequality (the so-called α-inequality):

(11) $$\begin{array}{c} \alpha \left(H\right)\leq \alpha^{*}\left(H\right), \end{array}$$

where α(H) is defined by Definition 12 and $\alpha^{*}\left(H\right)$ by Definitions 19 and 10, which is claimed to be a noncontextuality inequality too ([66], Results 1 and 2), although it should hold for any (hyper)graph ([48], p. 192), either non-binary or binary, i.e., for both NBMMPHs and BMMPHs.

Equation (11) certainly holds for general (hyper)graphs [48] (called the GLS inequality) where x(v) is a free variable, i.e., where $x\left(v\right)=w_{v}p\left(v\right)$, where p(v) is the probability of detecting vertex *v* and $w_{v}$ is the weight of this probability [66]. In the latter reference, it is stated that finding $\alpha^{*}$ is NP-hard. This is correct for the general GLS, but is it so for quantum measurements of MMPHs?

**Figure 4:**
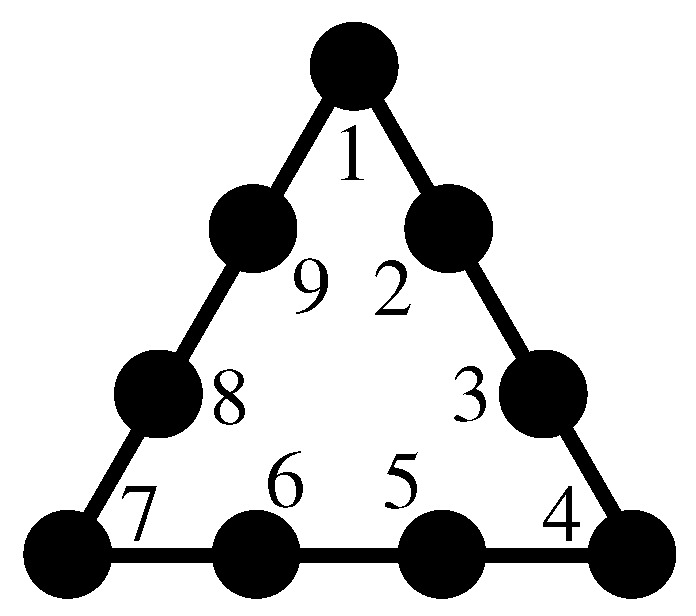
BMMPH 9-3.

Let us examine MMPH 9-3 given in Figure 4. For a free *x*, we have

LP[{−1,−1,−1,−1,−1,−1,−1,−1,−1},{{1,1,1,1,0,0,0,0,0},{0,0,0,1,1,1,1,0,0},{1,0,0,0,0,0,1,1,1}}, {{1,−1},{1,−1},{1,−1}}].

(LP is carried in *Mathematica* (LinearProgramming[]). Vectors/vertices are given as follows: {1,1,1,1,0,0,0,0,0} stands for the first hyperedge 1234, {0,0,0,1,1,1,1,0,0} for the second 4567, and {1,0,0,0,0,0,1,1,1} for the third 7891).

Out: ={0,1,0,0,1,0,0,1,0},

i.e., $\alpha^{*}=3$.

Since α=3, inequality (11) is satisfied.

However, for $x=p=\frac{1}{4}$, i.e., for quantum measurements, we obtain

LP[{−1,−1,−1,−1,−1,−1,−1,−1,−1},{{1,1,1,1,0,0,0,0,0},{0,0,0,1,1,1,1,0,0},{1,0,0,0,0,0,1,1,1}}, {{1,−1},{1,−1},{1,−1}}, {{$\frac{1}{4}$,1},{$\frac{1}{4}$,1},{$\frac{1}{4}$,1},{$\frac{1}{4}$,1},{$\frac{1}{4}$,1},{$\frac{1}{4}$,1},{$\frac{1}{4}$,1},{$\frac{1}{4}$,1},{$\frac{1}{4}$,1}}].

Out: ={$\frac{1}{4}$,$\frac{1}{4}$,$\frac{1}{4}$,$\frac{1}{4}$,$\frac{1}{4}$,$\frac{1}{4}$,$\frac{1}{4}$,$\frac{1}{4}$,$\frac{1}{4}$},

i.e., $\alpha^{*}=\frac{9}{4}=2.25$, which violates inequality (11).

See also the violations presented in the figure in Section 2.4.1.

In fact, such a regular/random distribution of, say, *n*-dim spin-$\frac{n-1}{2}$ MMPH systems exiting through the ports (vertices) of each of their gates/hyperedges (e.g., in a Stern–Gerlach device) is a well-known distribution of the spin projections of states of such systems. For systems composed of of several subsystems (e.g., several qubits, qutrits, and so on), a description is somewhat more complicated, but, in general, we can say that the systems satisfy the following postulate.

**Quantum Indeterminacy Postulate 1.**  *Quantum systems generated in an unknown (unprepared) pure state in an apparatus (e.g., a generalized Stern–Gerlach one), when exiting from it through one of the out-ports (channels, vertices) of their gates/hyperedges, have an equal probability of being detected on their exit ([67], Section 5-1).*

Hence, Equation (11) does not generally apply to quantum measurements, and the calculation of $\alpha^{*}$ is not of NP but of linear complexity. In fact, Equation (11) fails for many arbitrary quantum measurements, as shown below.

Measurements of a *k*-*l* set are carried out on hyperedges—hyperedge by hyperedge—and each hyperedge yields a single detection (click) corresponding to one of *n* vertices (vectors, states) contained in the hyperedges with a probability of $\frac{1}{n}$. This means that, for MMPHs whose hyperedges all contain *n* vertices, one builds the following statistics.

**Definition 21.**  **Raw data statistics** *for* MMPH*s whose all hyperedges contain n vertices (often adopted in the literature, e.g., ([9], Equation (2), [35], lines under Equation (2)), etc.), consist of assigning $\frac{1}{n}$ probability to each of the k vertices contained in the hypergraph (see Definition 3), independently of whether the vertices appear in just one hyperedge or in two or more of them.*

Such statistics do not appear to be satisfactory, though. Consider again 9-3 in Figure 4. The raw data statistics give us the sum of probabilities $\frac{9}{4}$. However, the vertices 1, 4, and 7 share two hyperedges and have multiplicity m=2. Thus, we actually have the sum of such calibrated probabilities being equal to $\frac{6}{4}+\frac{2\times 3}{4}=3$, i.e., we have a calibrated $\alpha^{*}=3$, which satisfies the inequality (11). We denote such a calibrated $\alpha^{*}$ as $\alpha_{p}^{*}$ and introduce the following more appropriate statistics:

**Definition 22.**  **Postprocessed MMPH data statistics** *are statistics for which*

*1.*  *vertex ‘v’ might share m(v) hyperedges;*
*2.*  *measurements are performed on n vertices v(j) contained in hyperedges ‘j’, j=1,…,l;*
*3.*  *the outcomes of measurements carried out on particular vertices v(j) in a particular hyperedge j might be dropped out of consideration, leaving us with κ(j) vertices in the hyperedge j;*
*4.*  *the probability of obtaining measurement data for each vertex within a hyperedge, after discarding the data for n−κ(j) dropped vertices, is $\frac{1}{\kappa (j)}$;*
*5.*  *the sum of all probabilities is, according to Equation *(7)*, equal to the size of the hypergraph, i.e., to the number of its hyperedges l.*

Such statistics yield the following theorem.

**Theorem 2.**  *Let variables x(v) from Definition *19* be the probabilities p(v), v∈V of detecting an event by YES-NO measurements at one of the out-ports (vertices) contained within a hyperedge of an n-dim* MMPH* H(V,E)=H(k-l). Each of $E_{j}\in E$, j=1,…,l hyperedges (gates) contains n vertices. The following holds:*

(12) $$\begin{array}{c} \sum_{v\in E_{j}}p\left(v\right)\leq 1,j=1,\ldots ,l. \end{array}$$

*They also satisfy the following:*

*(a) Under the raw data statistics (Definition *21*) assumption* [29,49,66]*, the sum of all probabilities is*

(13) $$\begin{array}{c} \sum_{v=1}^{k}p\left(v\right)=\frac{k}{n}=\alpha^{*}\left(k-l\right)=\alpha^{*}\left(H\right). \end{array}$$

*(b) Under the postprocessed data statistics (Definition *22*) assumption, i.e., under the assumption that every vertex v within an *MMPH *has $\frac{m(v)}{n}$ probability of being detected, the sum of all probabilities, according to the vertex-hyperedge Lemma 2, is*

(14) $$\begin{array}{c} \sum_{v=1}^{k}p\left(v\right)=\sum_{v=1}^{k}\frac{m(v)}{n}=l=\alpha_{p}^{*}\left(k-l\right)=\alpha_{p}^{*}\left(H\right) \end{array}$$

where $\alpha_{p}^{*}$ is called the postprocessed quantum fractional independence number.

**Figure 5:**
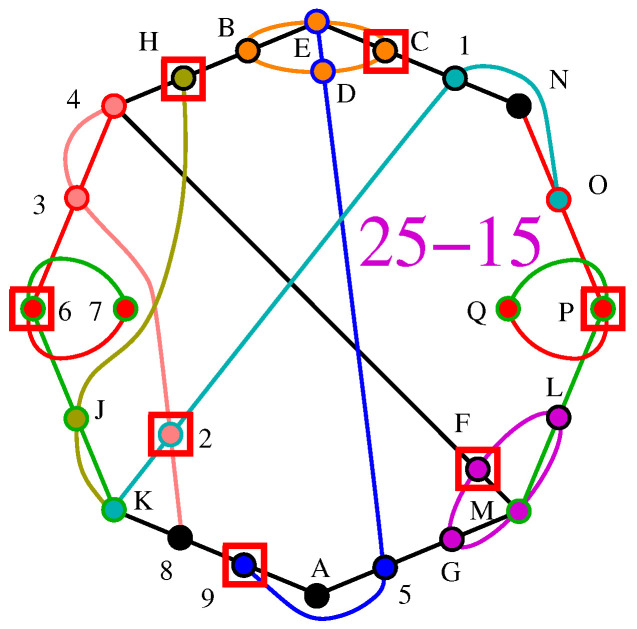
4-dim non-KS NBMMPH 25-15, a $\bar{\mathrm{subhypergraph}}$ of 26-15 ([3], Figure 6b); $\alpha =7>\alpha^{*}=\frac{25}{4}=6.25$; vertices contributing to α are red-squared; string and coordinatization are given in Appendix A.2.

**Figure 6:**
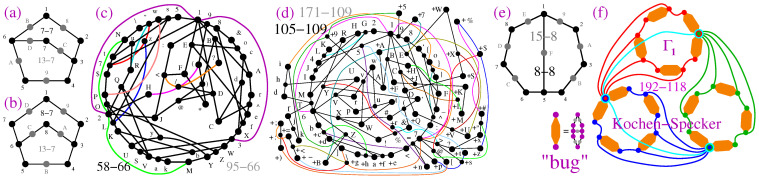
3-dim MMPHs. (**a**,**b**,**e**) Three smallest non-KS NBMMPHs; none can be substituted for the “bug” in $\Gamma_{1}$ in (**f**)—see text; (**c**) the smallest critical KS MMPH 95-66 obtained from golden ratio components (from 597-378 master) presented in the text (shown without m=1 vertices); (**d**) a much larger critical KS MMPH from the same master (shown without m=1 vertices); (**e**) 8-8 non-KS NBMMPH—see text; (**f**) a proper hypergraph encoding of the original Kochen and Specker design—see ([3], Figure 11); cf. improper encoding in ([52], Figure 1)—see text; m=1 vertices in (**a**,**b**,**e**) are depicted as grey dots.

Statement (a) implies that, in general, the α**-inequality**

(15) $$\begin{array}{c} HI_{cM}=\alpha \left(H\right)\leq \alpha^{*}\left(H\right)=\frac{k}{n} \end{array}$$

does not always hold for quantum mechanical measurements whose probabilities of detection within each hyperedge satisfy the condition given by Equation (12), i.e., under the Quantum Indeterminacy Postulate 1, as shown above in the LP analysis of MMPH 9-3 in Figure 5 and in arbitrarily many other NBMMPHs in any dimension (see ([3], Figure 6, Tables 2, 6, and 7). Note that $\alpha_{r}^{*}$ there [3] is equal to $\alpha^{*}$ here, according to the Quantum Indeterminacy Postulate 1. It is, therefore, not a reliable discriminator of contextual sets.

Statement (b) implies that the $\alpha_{p}^{*}$-inequality

(16) $$\begin{array}{c} HI_{cM}=\alpha \left(H\right)<\alpha_{p}^{*}\left(H\right)=l=HI_{q}, \end{array}$$

which follows from the vertex-hyperedge lemma (Equation (7)), is another form of the v-inequality (Equation (8)) and is therefore a noncontextuality inequality and a reliable discriminator of contextual sets.

Taken together, the MMPH e-inequality (Equation (9)), v-inequality (Equation (8)), and $\alpha_{p}^{*}$-inequality (Equation (16)) are genuine noncontextual inequalities and reliable discriminators of contextual sets, while the α-inequality (Equations (11) and (16)) is not, even though it is frequently presented as if it were, in the literature.

### 2.4. Generations of *KS* and *Non-KS MMPH*s in Dimensions 3 to 32

We provided a fairly exhaustive presentation of the generation of both KS and non-KS MMPHs in dimensions 3 to 8 in [3,4,46,47,50,54,55,56]. Here, we shall only present some new results and refer to particular previous findings in tables for the sake of completeness.

#### 2.4.1. 3-dim MMPHs

The smallest 3-dim non-KS MMPH (pentagon) [34] that we analyzed in [3] does not satisfy the basic requirement that all *n*-dim MMPHs in this paper should satisfy, i.e., that at least one of the hyperedges should have *n* vertices, meaning that at least one triple of orthogonal vectors should live in a 3-dim space. Thus, for example, the smallest non-KS NBMMPH 7-7, obtained by **M4-5**, smaller than the “bug” and shown in Figure 6a, does contain one hyperedge with three vertices.

The coordination of the filled 7-7 MMPH (182,234,495,5A6,6B1,3C7,6D7.) obtained by our programs is 1 = (1,−1,1), 2 = (1,0,−1), 3 = (1,0,1), 4 = (0,1,0), 5 = (1,0,0), 6 = (0,1,1), 7 = (1,1,−1), 8 = (1,2,1), 9 = (0,0,1), A = (0,1,−1), B = (2,1,−1), C = (−1,2,1), D = (2,−1,1). The next non-KS NBMMPHs obtained by the same methods are the 8-7 shown in Figure 6d and the “bug” shown in Figure 1.

These three MMPHs are interesting because, together with pentagons, hexagons, and heptagons, they prove that the “bug” plays a specific role in the structure of the original Kochen–Specker proposal, whose proper 192-118 MMP rendering is shown in Figure 6f (cf. ([52], Non-Figure 1)—where words are offered as unusual substitutes for hyperedges). If we substituted the aforementioned MMPHs for the bugs in the 192-118, they would transform it into a BMMPH.

The 8-8 shown in Figure 6e is obtained by **M2-3** from the 33–50 non-KS, which is a $\bar{\mathrm{subhypergraph}}$ (black dots) of the 69-50 shown in ([3], Figure 10e).

Thousands of other KS MMPHs in the real and complex 3-dim Hilbert space are presented in [50], while the non-KS ones derived from them are presented in [4].

Coordinatizations that we did not explore previously are those derived from variants of the *golden ratio* $\phi =(1+\sqrt{5})/2$. For instance, the following vector components: $\left\{0,\pm 1,\pm 2^{\frac{1}{2}}\phi^{\frac{3}{2}},\pm 2^{\frac{1}{2}}\phi^{-\frac{1}{2}},5^{\frac{1}{4}}\phi^{\frac{3}{2}},5^{\frac{1}{4}}\phi^{-\frac{3}{2}},\pm 2^{-1}\phi^{-1},2^{-1}5^{\frac{1}{4}}\phi^{\frac{1}{2}},\pm 2^{\frac{1}{2}}\phi^{-\frac{5}{2}},2^{-1}5^{\frac{1}{4}}\phi^{-\frac{5}{2}}\right\}$. They generate the 597-358 master, which in turn generates over 250 criticals, the smallest of which is shown in Figure 6c and one of the largest in Figure 6d. Note that, in ([50], Supp. Material, p. 3), we generated vectors, also partly based on the golden ratio, for the original Kochen and Specker’s design of their 192-118, since the equation they gave in [51] does not provide us with any definitive coordinatization (it contains two arbitrary parameters).

Table 1 gives an overview of the 3-dim MMPHs discussed above.

#### 2.4.2. 4-dim MMPHs

The 4-dim MMPHs are the most explored MMPHs in the literature over the last three decades.

In the beginning, several scientists spent five years seeking to find three KS MMPHs with {0,±1} components in three papers [68,69,70]. Fifteen years later, we are able to generate all 1233 KS MMPHs from these components in a few minutes on a PC from scratch [71].

**Figure 7:**
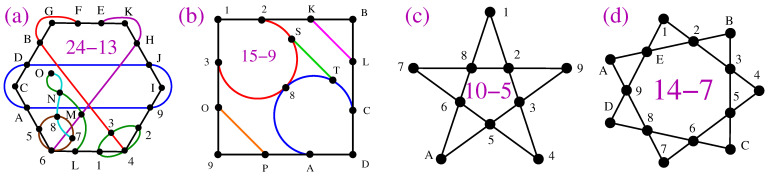
4-dim NBMMPHs. (**a**) A 24-13 KS MMPH critical that does not have a {0,±1} and is therefore not isomorphic to the 24-13 BMMPH from the aforementioned 4-dim 1233 class; its coordinatization is given in Appendix A.2; (**b**) the only critical non-KS NBMMPH obtained from 29-16 given in Appendix A.2; its string and coordinatization are given in Appendix A.2; (**c**) pentagram—see text; (**d**) heptagram—see text.

Later, large master sets based on serendipitous or intuitively found connections of KS hypergraphs with polytopes or Pauli operators were found [55,72,73,74,75]. Unfortunately, they only partly generated 4-dim KS sets, and their generation by means of these methods was neither automated nor generalized. Instead, this was achieved by an automated generation of MMPHs from basic components in [54,56]. Altogether, millions, if not billions, of 4-dim were generated in the last 15 years, so we only include several new MMPHs in Figure 7 and refer to all of them in Table 2.

The reason that we return to 4-dim MMPHs is that, originally, we were only interested in their structure and did not give the coordinatizations for them in our papers. Later on, we found a way to generate MMPHs directly from vector components and found several applications that required specifications of states/vectors. However, here, we give coordinatizations for several chosen MMPH masters generated from the simplest real and complex vector components, which has not been done previously. We also find particular symmetries in such generations. For instance, {0,±1,x} and {0,±x,1/x} might generate the same MMPH string, although, of course, not the same coordinatizations.

In New 4-dim MMPH Masters, Their Coordinatizations, and Their Distributions, we give strings and coordinatizations for MMPHs generated from the following vector components: {0,±1,ϕ} and $\left\{0,\pm \phi ,\frac{1}{\phi }\right\}$ yield 60-72, {0,±1,i} and {0,±i,1} yield 86-152, and {0,±i,±1} yield 92-185. The string and coordinatization of 86-152, as well as the distribution of the critical NBMMPHs contained in it, are given in New 4-dim MMPH Masters, Their Coordinatizations, and Their Distributions.

In fact, any new set of vector components might generate new master sets and classes of MMPHs. This is apparently misunderstood in [76], which after finding that “the vast majority of known examples have been found by computer search…without much insight in the sets generated”, offers a computer-free construction of an *infinite* family of KS sets in a 4-dim space instead. However, once the sets are generated, humans can offer any insight to them, and, once the vector components are defined ([76], Equation (1)), humans can generate MMPHs either by hand or by a computer. A computer might be much faster.

In the end, we consider star-like MMPHs: the 4-dim regular pentagram and heptagram (Schläfli symbols {5/2} and {7/2}) shown in Figure 7c,d. They are both KS MMPHs but apparently without coordinatizations, meaning that our programs written in C were run for days on a supercomputer, checking numerous vector components, and did not yield any results. We also used Mathematica to calculate the corresponding nonlinear equations on a supercomputer; after a month, it faced the 2 TB memory limitation. Note that the diagram in ([77], Figure 1) (which is graphically and misleadingly tantamount to Figure 7c) is not an MMPH (e.g., D1 and D2 are not orthogonal) and that, therefore, the states assigned to the vertices in that diagram do not represent a coordinatization of the MMPH pentagram. Moreover, the diagram {1,2,S2,f,S1,1}, which is called a pentagram in [77], is not a Schläfli {5/2} pentagram.

#### 2.4.3. 5- to 8-dim MMPHs

In higher dimensions, the simplest vector components {0,±1} allow us to obtain the most straightforward generation of MMPHs, although, in, e.g., the 6-dim space, the vector components {0,1,ω} generate smaller MMPHs than {0,±1}. Thus, we shall give examples of known vector components whenever available and/or interesting.

Moreover, in contrast to our previous generations, where we made use of mostly **M5**, here, in order to generate non-KS MMPHs, we chiefly use **M3**. In other words, to obtain non-KS MMPHs, we do not remove vertices with m>1 from KS MMPHs, as we mainly did in [4], but only those with m=1.

**Figure 8:**
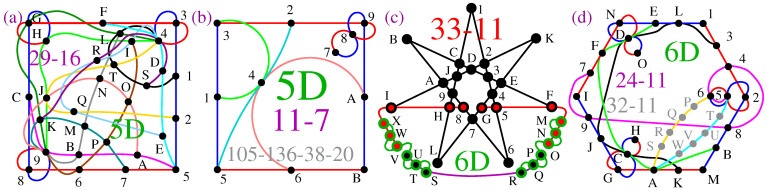
(**a**) One of the two smallest 5-dim criticals from the 105-136 KS master (the other was shown in ([50], Figure 2b); its string and coordinatization are given in Appendix A.3; (**b**) the smallest critical non-KS NBMMPH obtained from one of over 3000 38-20 KS MMPHs from the 105-136 class; the filled 11-7 is not necessarily a subhypergraph of the 38-20; their strings and coordinatization are given in Appendix A.3; (**c**,**d**) see text.

In the 5-dim space, {0,±1} components generate the 105-136 master, whose distribution we presented in [50]. There, we gave one of the two smallest KS MMPHs; in Figure 8a, we give the other (the string and coordinatization are given in Appendix A.3). It does not have vertices with m=1, and, overall, the class has a comparatively small number of NBMMPHs that contain m=1 vertices. Moreover, when they do, only a few of them generate small non-KS MMPHs. One such MMPH (11-7) is shown in Figure 8b. It is obtained from one of 38-20 KS MMPHs, but tracing the coordinatization down to the filled 11-7 and then to the 11-7 itself requires an algorithm whose calculation is more time-consuming than filling 11-7 up to 21-7 by MMPSHUFFLE and determining the coordinatization by VECFIND within less than 1 sec on a PC. The strings and coordinatization are given in Appendix A.3.

In the 5-dim space, a master MMPH obtained from {0,1,ω} vector components is a noncontextual BMMPH, in contrast to the 6-dim space, where these three components generate a star-like KS NBMMPH, which cannot be generated from {0,±1} components. (Star-like MMPHs exist only in even-dimensional spaces; we shall return to star-like constructions in Section 2.5.)

In the 6-dim space, vector components {0,1,ω} generate a 216-153 master, which contains over 15 million non-isomorphic KS MMPHs but just three critical KS MMPHs, 21-7, 27-9, and 33-11 (3*l*-*l*, odd l>5), the first two of which are shown in ([54], Figure A2). The third one, 33-11, which has not been previously presented in the star-like form, is given in Figure 8c, with its string and coordinatization in Appendix A.4. We can continue the 3*l*-*l* construction (see, e.g., the 39-13 in ([54], Figure A2)), but then we have to enlarge the set of vector component generators to $\left\{0,1,\omega ,\omega^{2}\right\}$ (ibid.).

The 6-dim {0,±1} vector components generate the 236-1216 KS master, which contains more than 3.7 million critical KS MMPHs ([55], Figure 12). A number of graphical representations of small critical MMPHs are given in ([55], Figure 11). Another example—32-11—is given in Figure 8d. It contains a non-KS NBMMPH 24-11. Their strings and coordinatization are given in Appendix A.4.

The 7- and 8-dim KS and non-KS NBMMPHs generated by {0,±1} vector components are extensively elaborated on in [4,47,50,55,56]. See Table 3.

#### 2.4.4. 9- to 32-dim MMPHs

Since the generation of MMPH masters from small vector components is an exponentially complex task, it is unfeasible to generate them in dimensions greater than eight. In [50], we successfully obtained a 9-dim master 9586-12068704 from the {0,±1} vector components. However, this master contains such a high number of BMMPHs that even a month-long run on a supercomputer did not yield any KS NBMMPHs. We were only able to extract non-KS NBMMPHs, since they are significantly more abundant.

Fortunately, our dimensional upscaling method **M8** does not scale in complexity with increasing dimensions. This allows us to generate MMPHS using a bottom-up approach, rather than relying on top-down generation from masters created from small vector components. In [47], we present the distributions of MMPHs obtained via this method across dimensions 9 to 16 and 27, as well as small instances of them. There, we also give a table of KS MMPHs for all these dimensions that are analogous to those in the tables above. In [4], we focus on generating non-KS MMPHs in dimensions 9 to 16. In [50], we generate 32-dim KS MMPHs from a five-qubit set derived in [78].

**Figure 9:**
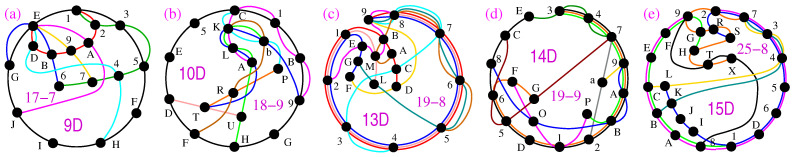
(**a**) A 9-dim non-KS 17-7 MMPH obtained from 19-8 in ([4], Figure 4b) by dropping m=1 vertices C and L and hyperedge EFI; note that the 19-8 stops being critical when L is removed, although it is an m=1 vertex; (**b**) a 10-dim non-KS 18-9 MMPH is presented via a circle of the largest hyperedges; (**c**–**e**) non-KS MMPHs presented via overlapping semi-circles (parts of hyperedges) featuring the δ-property—see text; the strings and coordinatizations of the MMPHs are given in [4].

In Figure 9, we offer a new graphical representation of some non-KS MMPHs from [4], providing a more comprehensive insight into their structure. Specifically, rather than depicting an MMPH by the largest loop formed by its hyperedges, we use circles or parts of circles to illustrate the hyperedges. The reader can compare Figure 9a–e with ([4], Figures 4b,c and 5a–c) to observe the difference. This presentation method allows us to visualize the so-called δ-feature [55] (in an *n*-dim space, hyperedges might share up to n−2 vertices; cf. Definition 2(4)) as overlapping semi-circles (cf. Figure 9c–e).

**Figure 10:**
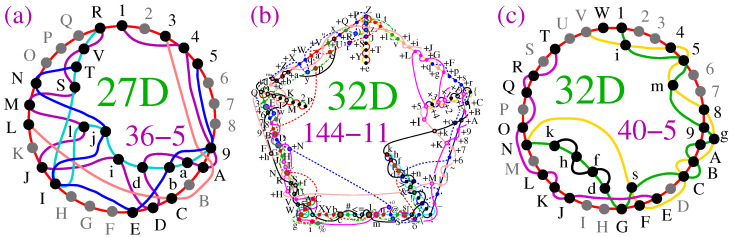
(**a**) A small 27-dim critical non-KS NBMMPHs obtained in this paper; its coordinatization can be derived from its master’s (141-16) vertices ([47], Appendix 14); (**b**) a 32 KS NBMMPH obtained in [55]; (**c**) a critical non-KS NBMMPH $\bar{\mathrm{subhypergraph}}$ obtained from it in this paper; see text for details.

Regarding higher-dimensional examples, Figure 10a presents a 27-dim non-KS MMPH $\bar{\mathrm{subhypergraph}}$ 36-5 obtained via **M2,3** from the 27-dim KS MMPH 141-16 master generated through method **M8** [47]. Consequently, there are gaps in the vertex numbering. While we could have closed these gaps, we chose not to do so in order to allow the reader to derive the coordinatization and reconstruct the missing vertices from the master 141-16 provided in [47]. As for the “missing” vertices—specifically dropped vertices with m=1—the circular hyperedge still contains m=1 (grey) vertices based on our principle of ensuring that all *n* vertices are included in at least one hyperedge. This principle enables the algorithms and programs to recognize a particular MMPH as belonging to an *n*-dim space. Furthermore, the automated reduction of 141-16 to the critical 36-5 ensures that the latter MMPH would remain an NBMMPH, meaning that it is still contextual, even if m=1 vertices from the circular hyperedge are removed. However, if we added one or more vertices to, for example, the pink hyperedge 3CL, the MMPH would cease to be contextual and would instead become a BMMPH. For further discussion of this property, see ([50], Supplementary Material p. 6).

In Figure 10c, we give a 32-dim non-KS MMPH 40-5 generated by **M2,3,5** from the 32-dim KS MMPH 144-11 obtained in [55]. All points that hold for the 27-dim $\bar{\mathrm{subhypergraph}}$ above hold for this one as well. For instance, if we added a vertex to the black hyperedge dfhk, the MMPH would lose its contextuality.

The list of non-KS NBMMPHs considered in this section is given in Table 4.

### 2.5. Applications

Possible applications of contextual sets/hypergraphs in higher dimensions do not face challenges in the generation of MMPHs but rather in their implementation. Apparently, the most feasible implementations would be those utilizing the angular momentum of photons in a holographic approach. Nevertheless, in this section, we will consider applications using a theoretical approach, addressing realistic implementation limitations only as necessary.

#### 2.5.1. Larger Alphabet

We extend the larger alphabet procedure discussed in [79]. A 4D KS “protection” for quantum key distribution (QKD) protocols was proposed in [26] based on a modification of the BB84 protocol outlined in [80]. A KS hypergraph with nine edges has been employed in this context. The protocol runs as follows: (i) Alice randomly picks one of the nine hyperedges (bases) and sends Bob a randomly chosen state (vertex) from that hyperedge; (ii) Bob randomly picks one of the nine hyperedges and measures the system received from Alice. Instead of qubits, we are working with ququarts, allowing the transfer of not 1 but 2 bits of information [79]. We can modify and generalize this QKD protocol to apply it to any *k*-*l* hypergraph (*k* vertices, *l* edges). However, this does not provide a quantum advantage to Alice and Bob. The reason is as follows.

**Figure 11:**
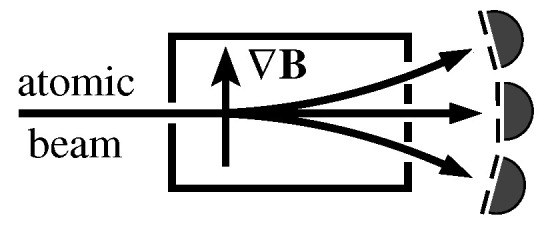
Stern–Gerlach experiment with spin-one atoms ([67], Figure 5-1).

Both KS and non-KS contextual sets (NBMMPHs) yield measurement outputs that differ from the predetermined measurement outputs that we would expect from classical sets that adhere to rules *(i)* and *(ii)* outlined in Definition 3. Let us illustrate this by means of the following simple Stern–Gerlach experiment. Quantum measurements, as illustrated in Figure 11, ideally always trigger one of the detectors positioned at each of the output ports, irrespective of whether the Stern–Gerlach devices are considered individually or networked (joined together) within an MMPH structure (see Quantum Indeterminacy Postulate 1). This contrasts with the classical counterparts. Individually, the classical devices produce the same outputs as the quantum ones, but, when networked and assumed to have predetermined output values, they should yield different results for at least one of the measurements of the devices/gates/hyperedges, meaning that, for this measurement, no detector should be triggered, i.e., all three vertices/outputs should be assigned a value of 0. Consequently, such networked classical devices are not feasible.

In other words, Alice and Bob can only achieve a quantum advantage if their eavesdropper, Eve, assumes that they communicate using outputs from networked classical devices. However, Eve will not assume this, since she knows that it is impossible due to the KS theorem. Instead, Eve will straightforwardly introduce fake messages for every hyperedge/gate, thus mimicking the quantum outputs and disregarding rules *(i)* and *(ii)* of Definition 3. Therefore, the “hybrid ququart-encoded quantum cryptography protected by Kochen–Specker contextuality” [26] is not truly “protected” against any Eve; it is simply another version of the BB84 protocol that offers no quantum advantage.

However, Alice and Bob can implement the following protocol in which Bob sends messages to Alice. We can assume that it is carried by photons carrying orbital angular momentum; over 100×100 entangled angular momentum dimensionality has been achieved experimentally [81]. It can also be implemented with higher-spin Stern-Gerlach devices [82].

- Alice picks up *n*-dim MMPHs and sends outputs from the gates/hyperedges of the chosen MMPHs to Bob in blocks; she can repeat sending from the same MMPHs or pick up new ones;
- Bob stores Alice’s sending in quantum memory;
- Alice informs Bob about which sending belonged to which hyperedge and from which MMPH over a classical channel, with a delay;
- Bob reads off each Alice’s sendings and sends them back to her, scrambled, over the quantum channel; scrambling codes transform Bob’s sendings into his messages but they are still undisclosed to Alice;
- Alice stores Bob’s sendings in quantum memory;
- Bob informs Alice of the scrambling code over a classical channel, with a delay;
- After an agreed number of exchanged blocks, they can transmit some messages over a classical channel to check whether Eve is in the quantum channel;
- After Alice has correlated the reflected sendings with the original ones with the help of Bob’s code, she learns how to measure each of them from the quantum memory and read off Bob’s message.

All steps in the protocol are completely automated. The probability that Eve might introduce correct sendings in the channel when Alice and Bob transfer the output of, say, the 32-dim MMPH with 11 hyperedges, shown in Figure 10b, is less than $3\times 10^{-17}$. Therefore, privacy amplification is hardly necessary.

**Figure 12:**
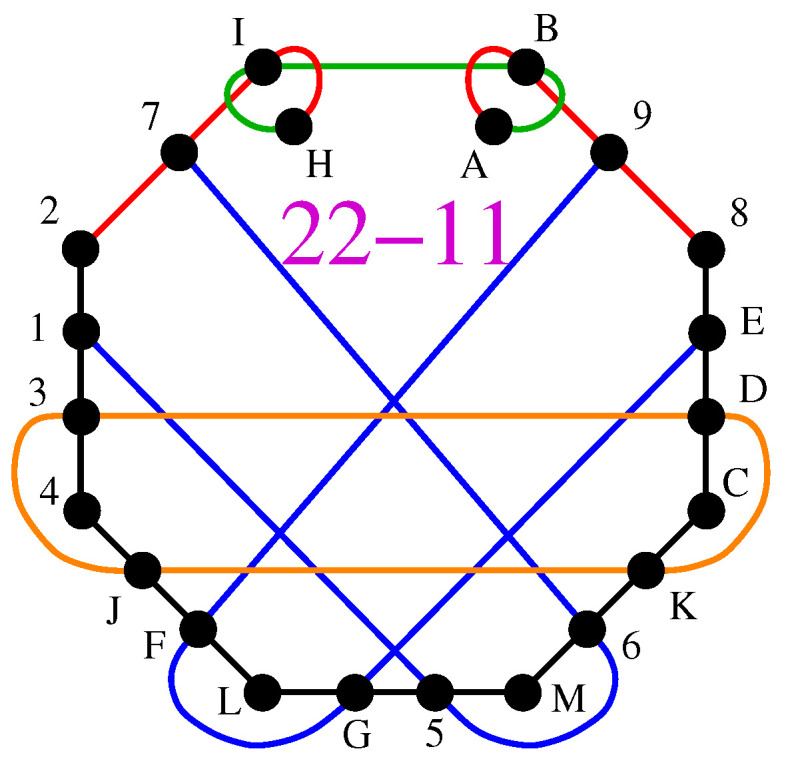
The 22-11 obtained from the 95-185 KS MPPH generated by vector components {0,±1,±i} or {0,±1,i}.

The advantage of the higher-dimensional protocol over the BB84 can be well exemplified by the fairly simple 22-11 KS MMPH shown in Figure 12.

The 22-11 cannot have a coordinatization based on {0,±1} vector components, but it can have this based on {0,±1,i}, {0,i,±1}, {0,±1,±i}, or {0,±1,2}, etc. Below are the string and coordinatization generated by the first two sets of vector components via the 86-152 master MMPHs (whose string, coordination, and distribution are given in New 4-dim MMPH Masters, Their Coordinatizations, and Their Distributions).

**22-11**      4312,27IH,HIBA,AB98,8EDC,CK6M,M5GL,LFJ4,1567,9FGE,J3DK.

1 = (0,0,0,1), 8 = (0,0,1,1), 9 = (0,0,1,−1), H = (0,0,1,i), I = (0,0,i,1), 2 = (0,1,0,0), J = (0,i,0,1), 5 = (0,1,i,0), 6 = (0,i,1,0), 7 = (1,0,0,0), M = (1,0,0,i), 3 = (1,0,i,0), 4 = (i,0,1,0), F = (1,1,i,i), C = (1,1,i,−i), D = (1,1,−i,i), G = (i,i,1,1), E = (1,−1,0,0), L = (1,−1,i,−i), K = (−1,1,i,i), A = (1,i,0,0), B = (i,1,0,0)

The coordinatization enables implementations via two qubits mounted on single photons by means of linear and circular polarization and orbital angular momentum. To see this, let us first define the photon qubit states:

(17) $$\begin{array}{ccc} & & |H\rangle ={\left(\begin{array}{c} 1\\ 0 \end{array}\right)}_{1},\;|V\rangle ={\left(\begin{array}{c} 0\\ 1 \end{array}\right)}_{1},\;|D\rangle =\frac{1}{\sqrt{2}}{\left(\begin{array}{c} +1\\ 1 \end{array}\right)}_{1},\;|A\rangle =\frac{1}{\sqrt{2}}{\left(\begin{array}{c} -1\\ 1 \end{array}\right)}_{1},\;|R\rangle =\frac{1}{\sqrt{2}}{\left(\begin{array}{c} 1\\ +i \end{array}\right)}_{1},\\ & & |L\rangle =\frac{1}{\sqrt{2}}{\left(\begin{array}{c} 1\\ -i \end{array}\right)}_{1},\;|2\rangle ={\left(\begin{array}{c} 1\\ 0 \end{array}\right)}_{2},\;|-2\rangle ={\left(\begin{array}{c} 0\\ 1 \end{array}\right)}_{2},\;|h\rangle =\frac{1}{\sqrt{2}}{\left(\begin{array}{c} 1\\ 1 \end{array}\right)}_{\;2},\;|v\rangle =\frac{1}{\sqrt{2}}{\left(\begin{array}{c} 1\\ -1 \end{array}\right)}_{\;2}, \end{array}$$

where H,V are horizontal and vertical, D,A are diagonal and anti-diagonal, and R,L are right and left circular polarizations, while ±2 are Laguerre–Gauss modes carrying ±2ℏ units of orbital angular momentum (OAM) and h,v are their ± superpositions, respectively. Indices ‘1’ and ‘2’ refer to the first and second qubits mounted on the system (single photon), respectively.

These qubit states build the hyperedge gates as tensor products, e.g.,

(18) $$\begin{array}{ccc} & & |G\rangle =\frac{1}{\sqrt{2}}\left(\begin{array}{c} i\\ i\\ 1\\ 1 \end{array}\right)=\frac{1}{2}\left(\begin{array}{c} i\left(\begin{array}{c} 1\\ 1 \end{array}\right)\\ 1\left(\begin{array}{c} 1\\ 1 \end{array}\right) \end{array}\right)=\frac{1}{\sqrt{2}}{\left(\begin{array}{c} i\\ 1 \end{array}\right)}_{1}⊗\frac{1}{\sqrt{2}}{\left(\begin{array}{c} 1\\ 1 \end{array}\right)}_{2}=-{i|L\rangle }_{1}{|h\rangle }_{2},\\ & & |L\rangle =\frac{1}{\sqrt{2}}\left(\begin{array}{c} 1\\ -1\\ i\\ -i \end{array}\right)=\frac{1}{2}\left(\begin{array}{c} 1\left(\begin{array}{c} 1\\ -1 \end{array}\right)\\ i\left(\begin{array}{c} 1\\ -1 \end{array}\right) \end{array}\right)=\frac{1}{\sqrt{2}}{\left(\begin{array}{c} 1\\ i \end{array}\right)}_{1}⊗\frac{1}{\sqrt{2}}{\left(\begin{array}{c} 1\\ -1 \end{array}\right)}_{2}={|R\rangle }_{1}{|v\rangle }_{2} \end{array}$$

Some states (e.g., 5) require rather involved manipulation (including superpositions of tensor products).

Fortunately, in a large alphabet quantum cryptography, we can limit ourselves to {0,±1} vector components in any higher dimension. It is only in the 4-dim space that there are no more than six critical MMPHs with a coordinatization based on {0,±1} vector components.

Here, with one of the smallest 4-dim MMPHs, the states build eleven hyperedges/gates, which cause Eve’s probability of correctly guessing all states to be less than $10^{-6}$.

Notice the orthogonality of complex vectors, e.g., A$\cdot B^{*}=\left(1,i,0,0\right)\cdot {\left(i,1,0,0\right)}^{*}=\left(1,i,0,0\right)\cdot \left(-i,1,0,0\right)=0$.

Note that the generation of the states from Alice and Bob’s devices is genuinely random (quantum randomness) [83]. Moreover, Bob scrambles Alice’s sending through a quantum random number generator.

One can object that the protocol could be implemented using other quantum sets and not just MMPHs. While this is true, the abundance and automated generation of MMPHs of any size and dimension make them the most favourable candidates for large alphabet communication in higher dimensions.

#### 2.5.2. Oblivious Communication Protocol

Communication in a system with no dimensional bound and with some information about the sender’s input unrevealed, i.e., oblivious communication, is presented in [28].

However, Alice and Bob can have a quantum advantage only if their eavesdropper (Eve) assumes that they communicate using outputs obtained from networked classical devices. Since they do not, the oblivious communication protocol needs to be modified so as to follow the protocol proposed in Section 2.5.1. Then, the protocol could utilize any KS MMPH in any dimension.

#### 2.5.3. Generalized Hadamard Matrices

**Figure 13:**
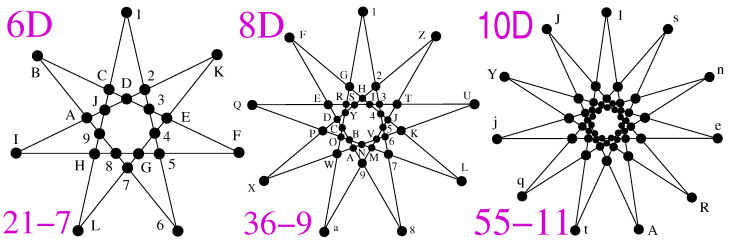
Star-like $\frac{n}{2}\left(n+1\right)$-(n+1), (*n* even), *n*-dim MMPHs related to *S*-*H* matrices; see text.

Most quantum computation algorithms are based on the Fourier transform, of which the Hadamard (*H*) transform is a special case. Recently, the *S* class of *H* matrices, known as *S*-*H* matrices in $C^{n}$, with *n* being even, has been designed to prove the existence of KS hypergraphs in an *n*-dim space [84]. Our method generates any of these KS hypergraphs, which are all found to be star-like (see Figure 13). Inversely, it allows us to generate the elements of the corresponding *S*-*H* matrices. The *S*-*H* matrices depend on the following theorem.

**Theorem 3**  (Lisoněk 2019)**.** *Suppose that there exists an S-H matrix of order n (n even); then, there exists a* KS *hypergraph k-l in $C^{n}$ such that $k=\left(\frac{n+1}{2}\right)$ and l=n+1.*

In [84], Lisoněk defines a KS hypergraph in $C^{n}$ (Definition 1). By Definition 2.1, he defines an *S*-*H* matrix and, in Definition 2.2, a generalized Hadamard matrix. Via Theorem 3, he connects a KS hypergraph and a corresponding *S*-*H* matrix. The proof provides an algorithm for a mutual one-to-one mapping of their elements in any even dimension. However, the details are beyond the scope of the present paper, and we direct the reader to ref. [84]. Definitions 1, 2.1, 2.2 and Theorem 3 above are from ref. [84].

Only the 6D 21-7 KS hypergraph was known to Lisoněk in [84], i.e., only one particular *S*-*H* matrix, aside from the *existence* of all of them. Our method generates any of these KS hypergraphs together with their coordinatization (although the time required to generate the coordinatization rises exponentially with the dimension, the time required to generate MMPHs themselves does not). Inversely, as is the core of this application, it gives the elements of the corresponding *S*-*H* matrices. It further demonstrates that all the corresponding KS hypergraphs are star-like; see Figure 13. This feature clarifies why *n* has to be even: one cannot draw a regular star with the Schläfli symbol {*n*+1/$\frac{n}{2}$} in odd-dimensional spaces because $\frac{n}{2}$ has to be an integer. The 6,- 8-, and 10-dim star strings and coordinatizations are given in ([47], Appendix).

#### 2.5.4. Stabilizer Operations

**Figure 14:**
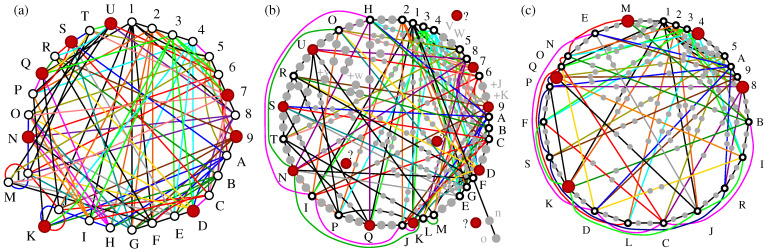
(**a**) Stabilizer non-KS 30-108 NBMMPH [29]: $\alpha =8<\alpha^{*}=9$; ([3], Section 5.4); (**b**) filled (**a**): noncritical 232-108 KS MMPH violates the α-inequality: $\alpha =101>\alpha^{*}=58$ (some red vertices that might contribute to α are indicated by “?”; so, e.g., the red dot near vertex n at the right bottom of the figure indicate that n might contribute to α); (**c**) critical 152-71 that violates the α-inequality: $\alpha =64>\alpha^{*}=38$; with m=1 (grey) vertices dropped, we have $\bar{\mathrm{subhypergraph}}$ 24-71, which satisfies the α-inequality: $\alpha =5<\alpha^{*}=6$.

Stabilizer operations entail MMPHs [3,29]. In particular, in [29], a graph is obtained that has a one-to-one correspondence with the non-KS 30-108 NBMMPH, shown in Figure 14a and obtained in ref. ([3], Section 5.4). Its filled MMPH 232-108, shown in Figure 14b, is a KS NBMMPH, but the latter is not critical. It contains just one critical KS MMPH: the 152-71 one. Notice that it too violates the α-inequality $\alpha =64>\alpha^{*}=38$ ([3], Table 5) but is nevertheless contextual. When its m=1 vertices are dropped, it becomes the 24-71 non-KS NBMMPH shown in Figure 14c, which satisfies the α-inequality: $\alpha =5<\alpha^{*}=6$. Thus, notwithstanding the α-inequality, all these MMPHs are NBMMPHs, i.e., contextual, and therefore might provide a path toward finding simpler stabilizer operations by reducing the original noncritical stabilizer NBMMPHs to smaller, critical ones.

## 3. Discussion

We present a survey and further development, as well as promising applications, of quantum contextual sets represented by a type of hypergraph called McKay–Megill–Pavičić hypergraphs (MMPHs; Definition 2). Such a representation is universal as it can be demonstrated that most presentations of contextual sets—including polytopes, Lie groups, operators, projectors, vectors, and states—have a one-to-one correspondence with MMPHs [3]. A good outline of such alternative methods is given in ref. [56].

In this paper, we focus on two methods of obtaining contextual MMPHs: the vector generation of MMPHs from basic vector components via the **M1** method (see Section 2.2) and the dimensional upscaling of MMPHs via the **M8** method.

The former method involves creating an internal list of all possible non-zero vectors containing simple vector components like {0,±1} or {0,±i}. From this list, a program (VECFIND) finds a set of all possible mutually orthogonal vector *n*-tuples of vectors and, from them, it generates an MMPH with hyperedges corresponding to mutually orthogonal *n*-tuples, which is called a master MMPH. Contextual MMPHs, i.e., NBMMPHs (Definition 3), are then filtered out by the program STATES01. These NBMMPHs allow us to obtain a class (Definition 6) of critical (Definition 5) KS MMPHs (Theorem 1) as well as the non-KS MMPHs (Definition 16) via **M3** and **M5**.

The latter method consists of using comparatively small KS MMPHs obtained with the help of the former method to build KS MMPHs in higher dimensions, which then allow us to recursively obtain KS MMPHs in increasingly higher dimensions, since the complexity of the method does not scale with dimension. This method works by combining two KS MMPHs from lower dimensions so as to allow the number of unique vertices in the new combined MMPH to be minimized and filter out KS MMPHs. We start with $k_{1}$ vectors in the $n_{1}$-dim space and $k_{2}$ vectors in the $n_{2}$-dim space and construct an MMPH with *n*-dim vectors, where $n<n_{1}+n_{2}$—vectors of parent MMPHs are extended with enough vector components 0 to reach dimension *n*. In the first MMPH, 0s are appended at the ends of the existing vectors. In the second MMPH, the *n*-$n_{2}$-dim 0s are prefixed at the start of the vectors, which ensures that the new MMPH has non-zero vector components in all *n* cardinal directions in the space. Since the vector components {0,±1} are used, the number of vectors *k* of the *n*-dim space is significantly smaller than $k_{1}+k_{2}$ for most parent KS MMPHs, and almost all of them are KS MMPHs.

In Section 2.4.1, Section 2.4.2 and Section 2.4.3, we explore generations of MMPHs in dimensions three to eight from simple vector components, such as {0,±1}, and discuss specific new instances that have not previously been considered. We distinguish two main groups of contextual MMPHs: Kochen–Specker (KS) MMPHs (Definition 15) and non-KS MMPHs (Definition 16). The KS MMPHs are generated from large master MMPHs and yield non-KS MMPHs. However, the generation of these masters and subsequent smaller MMPHs from them is a task of exponential complexity, which limits the methods of obtaining them (Methods **M1,2**, Section 2.2) to dimension eight. To overcome this limitation, we have developed methods **M7** and **M8**, whose complexity does not scale with dimension, and we elaborate on them in dimensions 9 to 32 in Section 2.4.4 while presenting a new graphical representation of MMPHs that offers a more comprehensive insight into their structure. The minimal number of hyperedges in non-KS MMPHs repeatedly varies between 8 (odd dimensions) and 9 (even dimensions). For KS NBMMPHs, this number fluctuates between 9 and 16. We provide lists of the NBMMPHs across all dimensions in Table 1, Table 2, Table 3 and Table 4.

The extensive range of MMPHs has allowed us to identify several features that they exhibit, as well as the relations and quantifications of contextuality that they support. In Section 2, we present several lemmas and theorems related to these features, along with two types of statistics (Definitions 21 and 22). We also prove that the α-inequality between the independence number α (Definition 12) and the fractional independence (packing) number $\alpha^{*}$ (Definition 10), expressed as $\alpha \leq \alpha^{*}$ (Equation (11)), does not always hold for quantum measurements of MMPH states. The reason is that, in quantum measurements, all vertices of a hyperedge must be assigned an equal and constant probability when measured, which leads us to the Quantum Indeterminacy Postulate 1 and two types of statistics, *raw* MMPH *data statistics* (Definition 21) and *postprocessed* MMPH *data statistics* (Definition 22), as well as to two noncontextual inequalities, the *hyperedge inequality* (Equation (9)) and the *postprocessed quantum fractional independence number inequality* (Equation (16)), which serve as reliable discriminators of contextual MMPHs, in contrast to the α-inequality, which does not. In Figure 5, we show several KS and non-KS MMPHs that violate the α-inequality. Hence, the α-inequality in, e.g., [29,66] does not *reveal* contextuality but is fortuitously satisfied for the MMPHs that might already be known as contextual, i.e., as NBMMPHs, what can be reliably verified with the help of our programs STATES01 and ONE—cf. Figure 14a,b—by processing measurement data.

In Section 2.5, we propose four possible applications of contextual MMPHs.

In Section 2.5.1, we consider larger alphabet quantum key distribution (QKD) protocols in higher dimensions. We note that conventional quantum contextual protocols, as proposed in sources like [26], do not offer a quantum advantage over the corresponding classical protocols, due to the impossibility of the existence of such competitive classical protocols by virtue of the Kochen–Specker theorem. Therefore, we design a protocol that does provide a quantum advantage over possible classical competitors such as BB84, even when expanded to larger alphabets. The protocol relies on the Quantum Indeterminacy Postulate 1 and genuine quantum randomness.

In Section 2.5.2, we extend the previous protocol to a quantum oblivious communication protocol in higher dimensions.

In Section 2.5.3, we examine the *S* class of the Hadamard transform, which is a special case of the Fourier transform that underlies most quantum computation algorithms. We establish a one-to-one correspondence between the elements of the *S* class and star-like KS MMPHs, thereby allowing us to derive elements of an S matrix from a corresponding star-like KS MMPH.

In Section 2.5.4, we explore the application of NBMMPHs in simplifying stabilizer operations. Specifically, we consider a recent derivation of a graph from a stabilizer operation and demonstrate that translating this graph into a non-KS MMPH allows for the addition of m=1 vertices to form a KS MMPH. The resulting critical KS MMPH and its underlying non-KS MMPH (with m=1 vertices removed) are significantly smaller than the original versions. This approach could lead to a reduced set of stabilizer operations and facilitate the discovery of simpler error correction codes.

## 4. Methods

The methods that we employ to manage quantum contextual sets are based on algorithms and programs developed within the MMP language, including VECFIND, STATES01, MMPSTRIP, MMPSHUFFLE, SUBGRAPH, LOOP, SHORTD, and ONE, as referenced in [3]. These resources are freely accessible at http://puh.srce.hr/s/Qegixzz2BdjYwFL (accessed on 30 December 2024). MMPHs can be visualized as hypergraph figures, consisting of dots and lines, and can also be represented as strings of ASCII characters. This representation enables simultaneous processing of billions of MMPHs using supercomputers and clusters. To facilitate the processing, we have developed additional dynamical programs to manage and parallelize tasks involving any number of MMPH vertices and edges.

## 5. Conclusions

In summary, based on elaborations of KS and non-KS contextual sets presented in the literature and further developed in this paper, we have developed methods for the generation of contextual sets, revealed their properties, and designed their applications across any dimension. We provide examples in dimensions up to 32 and give reliable discriminators of their contextuality. A more detailed summary of the achieved results is given in Section 3.

## Figures and Tables

**Table 1:** 3-dim NBMMPHs obtained by methods **M1-2** and **M4-5**; ‘*’ indicates that the MMPH is a KS; ‘†’ indicates that the MMPH is a noncontextual BMMPH; ‘‡’ indicates that the MMPH is a non-KS contextual NBMMPH.

dim	Master	No. of Non-Isom Criticals	Methods	Smallest KS and Non-KS Criticals	Vector Components	References
3-dim	13-7 ^†^	1	**M4-5**	7-7 ‡	{0,±1,2}	see text
	13-7 ^†^	1	**M4-5**	8-7 ‡	{0,±1,2}	see text
	97-64	1	**M1-2**	49-36 * (33-36 ‡)	{0,±1,±2,5}	[3]
	81-52	1	**M1-2**	57-40 * (33-50 ‡)	$\left\{0,\pm 1,\sqrt{2},\pm 3\right\}$	[3]
	169-120	3	**M1-2**	69-50 * (33-50 ‡, 8-8 ‡)	$\;\;(0,\pm \omega ,2\omega ,\pm \omega^{2},2\omega^{2}\}$	[3], see text
	597-358	3	**M1-2**	95-66 * (58-66 ‡)	golden ratio—see text	see text

**Table 2:** 4-dim NBMMPHs obtained as described in the text; ‘‡’ indicates that the MMPH is a non-KS contextual NBMMPH. The first critical is given in Appendix A.2; others are given in Figure 7 and Appendix A.2; “none ?” means that there might not be any coordinatization.

dim	Master	No. of Non-Isom Criticals	Methods	KS and Non-KS Criticals	Vector Components	References
4-dim	86-152	>8 millions	**M1-6**	10-7 ‡, 15-9 ‡, 18-9, 24-13	{0,±1,i}	see text
	92-185	600,000	**M1-6**	18-9 (smallest)	{0,±1,±i}	[55,56]
	888-1080	>1.5 billions	**M1-6**	18-9 (smallest)	$\left\{0,\pm \phi ,\frac{1}{\phi }\right\}$	[55,56]
	400-1012	>250,000	**M1-6**	18-9 (smallest)	$\left\{0,\pm 1,\pm \omega ,\pm \omega^{2}\right\}$	[55,56] and here
	10-5	1	**M6**	10-5	none ?	see text
	14-7	1	**M6**	14-7	none ?	see text

**Table 3:** NBMMPHs obtained by methods **M1-3** and **M8**; ‘*’ indicates that the MMPH is a KS ‘‡’ indicates that the MMPH is a non-KS contextual NBMMPH.

dim	Master	No. of Non-Isom Criticals	Methods	Small MMPHs	Vector Components	References
5-dim	105-136	>27.8 millions	**M1**	29-16 *	{0,±1}	see text
	38-20	-	**M2-3**	5-11 ‡	{0,±1}	see text
6-dim	216-153	3	**M1**	33-11 *	{0,1,ω}	see text
	236-1216	>3.7 millions	**M2-3**	32-11† (24-11 ‡)	{0,±1}	see text
7-dim	47-176	>1 million	**M2,8**	34-14 *	{0,±1}	[47,50]
	805-9936	>42,800	**M1,3**	14-18 ‡	{0,±1}	[4]
8-dim	3280-1361376	>7 millions	**M1-2**	36-9 * (15-9 ‡)	{0,±1}	[4,50]

**Table 4:** The smallest critical non-KS MMPHs obtained via **M2**, **M7**, and **M8**. Notice the steady fluctuation in the number of hyperedges—the minimum complexity of NBMMPHs does not grow with the dimension.

dim	Smallest Critical Non-KS NBMMPHs	Master	Vector Components
9-dim	17-7	47-16	{0,±1}
10-dim	18-9	50-15	{0,±1}
11-dim	19-8	50-14	{0,±1}
12-dim	19-9	52-9	{0,±1}
13-dim	19-8	63-16	{0,±1}
14-dim	19-9	66-15	{0,±1}
15-dim	25-8	66-14	{0,±1}
16-dim	22-9	70-9	{0,±1}
27-dim	36-5	141-16	{0,±1}
32-dim	40-5	144-11	{0,±1}

## Data Availability

All data additional to those given in Appendix A can be obtained directly from the author.

## Abbreviations

    The following abbreviations are used in this manuscript:

MMPH	McKay–Megill–Pavičić hypergraph (Definition 2)
NBMMPH	Non-binary McKay–Megill–Pavičić hypergraph (Definition 3)
BMMPH	Binary McKay–Megill–Pavičić hypergraph (Definition 4)
KS	Kochen–Specker (Definition 15)
non-KS	Non-Kochen–Specker (Definition 16)
**M1, M2, …, M8**	Methods 1, 2, …, 8 (Section 2.2)

## Appendix A. ASCII Strings of Non-KS MMPH Classes and Their Masters and Supermasters

Below, we give the strings and coordinatizations of all MMPHs referred to in the main body of the paper. The first hyperedges in a line of a critical NBMMPH often correspond to the largest loops in the figures.

### Appendix A.1. 3-dim MMPHs

**8-7** (KS “bug”) 123,34,45,567,78,81,26.

**13-7** (filled **8-7**) 123,394,4A5,567,7B8,8C1,2D6. 1 = (0,0,1), 2 = (0,1,0), 3 = (1,0,0), 4 = (0,1,1), 5 = (1,1,−1), 6 = (1,0,1), 7 = (−1,2,1), 8 = (2,1,0), 9 = (0,1,−1), A = (2,−1,1), B = (1,−2,5), C = (1,−2,0), D = (1,0,−1).

**25-16** (BMMPH) 123,345,567,789,9AB,BCD,DEF,FGH,HI1,1JK,KLA,JM7,JPD,HN9, 3OB,FL5. 1 = (0,0,1), 2 = ($\sqrt{2}$,−1,0), 3 = (1,$\sqrt{2}$,0), 4 = ($\sqrt{2}$,−1,−3), 5 = ($\sqrt{2}$,−1,1), 6 = ($\sqrt{2}$,3,1), 7 = (−1,0,$\sqrt{2}$), 8 = ($\sqrt{2}$,−3,1), 9 = ($\sqrt{2}$,1,1), A = (0,1,−1), B = ($\sqrt{2}$,−1,−1), C = ($\sqrt{2}$,3,−1), D = (1,0,$\sqrt{2}$), E = ($\sqrt{2}$,−3,−1), F = ($\sqrt{2}$,1,−1), G = ($\sqrt{2}$,1,3), H = (−1,$\sqrt{2}$,0), I = ($\sqrt{2}$,1,0), J = (0,1,0), K = (1,0,0), L = (0,1,1), M = ($\sqrt{2}$,0,1), N = ($\sqrt{2}$,1,−3), O = ($\sqrt{2}$,−1,3), P = ($\sqrt{2}$,0,−1).

**95-66** 123,145,167,189,1AB,1CD,1EF,2GH,2IJ,2KL,2MN,2OP,2QR,ST3,SUV,WX3, WYZ,Wab,Xcd, Xef,KgU,Khb,MiY,Mjk,lQa,mEe,nEo,pAc,qAr,stu,svL,sw5,xQy,z!L, z"7,t#N,t$7,8%&,8’f,()*, (-9,(/H,:;9,:<J,=)B,>?B,)@J,?/<,[∖N,]C^,_Cd,‘OV, {OZ,∖w",|"R, }<F,∼*F,+1/D,+2@D,+3Ru, +4wP,+5$P,kaV,yYU,re^,co&. 1 = (0,0,1), 2 = (0,1,0), S = (0,1,$-2^{\frac{1}{2}}\phi^{\frac{3}{2}}$), W = (0,1,$2^{\frac{1}{2}}\phi^{-\frac{1}{2}}$), T = (0,$2^{\frac{1}{2}}\phi^{\frac{3}{2}}$,1), X = (0,$-2^{\frac{1}{2}}\phi^{-\frac{1}{2}}$,1), 3 = (1,0,0), G = (1,0,$2^{\frac{1}{2}}\phi^{\frac{3}{2}}$), I = (1,0,$-2^{\frac{1}{2}}\phi^{-\frac{1}{2}}$), K = (1,0,$5^{\frac{1}{4}}\phi^{\frac{3}{2}}$), M = (1,0,$5^{\frac{1}{4}}\phi^{-\frac{3}{2}}$), i = ($2^{-1}5^{\frac{1}{4}}\phi^{-\frac{5}{2}}$,$-2^{\frac{1}{2}}\phi^{-\frac{5}{2}}$,$-2^{-1}\phi^{-1}$), l = ($2^{-1}5^{\frac{1}{4}}\phi^{-\frac{5}{2}}$,$2^{\frac{1}{2}}\phi^{-\frac{5}{2}}$,$2^{-1}\phi^{-1}$), m = ($2^{-1}5^{\frac{1}{4}}\phi^{-\frac{5}{2}}$,$2^{-1}\phi^{-1}$,$-2^{\frac{1}{2}}\phi^{-\frac{5}{2}}$), n = ($2^{-1}5^{\frac{1}{4}}\phi^{-\frac{5}{2}}$,$2^{-1}\phi^{-1}$,$2^{\frac{1}{2}}\phi^{-\frac{1}{2}}$), 4 = (1,$2^{\frac{1}{2}}\phi^{\frac{3}{2}}$,0), p = ($2^{-1}5^{\frac{1}{4}}\phi^{-\frac{5}{2}}$,$-2^{-1}\phi^{-1}$,$2^{\frac{1}{2}}\phi^{-\frac{5}{2}}$), q = ($2^{-1}5^{\frac{1}{4}}\phi^{-\frac{5}{2}}$,$-2^{-1}\phi^{-1}$,$-2^{\frac{1}{2}}\phi^{-\frac{1}{2}}$), s = (1,$2^{\frac{1}{2}}\phi^{\frac{3}{2}}$,$5^{\frac{1}{4}}\phi^{\frac{3}{2}}$), j = ($2^{-1}5^{\frac{1}{4}}\phi^{-\frac{5}{2}}$,$2^{\frac{1}{2}}\phi^{-\frac{1}{2}}$,$-2^{-1}\phi^{-1}$), x = ($2^{-1}5^{\frac{1}{4}}\phi^{-\frac{5}{2}}$,$-2^{\frac{1}{2}}\phi^{-\frac{1}{2}}$,$2^{-1}\phi^{-1}$), 6 = (1,$-2^{\frac{1}{2}}\phi^{-\frac{1}{2}}$,0), z = (1,$-2^{\frac{1}{2}}\phi^{-\frac{1}{2}}$,$5^{\frac{1}{4}}\phi^{\frac{3}{2}}$), t = (1,$-2^{\frac{1}{2}}\phi^{-\frac{1}{2}}$,$5^{\frac{1}{4}}\phi^{-\frac{3}{2}}$), 8 = (1,$5^{\frac{1}{4}}\phi^{\frac{3}{2}}$,0), ( = (1,$5^{\frac{1}{4}}\phi^{\frac{3}{2}}$,$2^{\frac{1}{2}}\phi^{\frac{3}{2}}$), : = (1,$5^{\frac{1}{4}}\phi^{\frac{3}{2}}$,$-2^{\frac{1}{2}}\phi^{-\frac{1}{2}}$), = = ($2^{-1}\phi^{-1}$,$2^{-1}5^{\frac{1}{4}}\phi^{-\frac{5}{2}}$,$2^{\frac{1}{2}}\phi^{-\frac{5}{2}}$), > = ($2^{-1}\phi^{-1}$,$2^{-1}5^{\frac{1}{4}}\phi^{-\frac{5}{2}}$,$-2^{\frac{1}{2}}\phi^{-\frac{1}{2}}$), A = (1,$5^{\frac{1}{4}}\phi^{-\frac{3}{2}}$,0), # = ($2^{-1}\phi^{-1}$,$2^{\frac{1}{2}}\phi^{-\frac{5}{2}}$,$2^{-1}5^{\frac{1}{4}}\phi^{-\frac{5}{2}}$), ) = (1,$5^{\frac{1}{4}}\phi^{-\frac{3}{2}}$,$-2^{\frac{1}{2}}\phi^{-\frac{1}{2}}$), - = ($2^{-1}\phi^{-1}$,$2^{-1}5^{\frac{1}{4}}\phi^{\frac{1}{2}}$,$-2^{\frac{1}{2}}\phi^{-\frac{1}{2}}$), ; = ($2^{-1}\phi^{-1}$,$2^{-1}5^{\frac{1}{4}}\phi^{\frac{1}{2}}$,$2^{\frac{1}{2}}\phi^{\frac{3}{2}}$), ? = (1,$5^{\frac{1}{4}}\phi^{-\frac{3}{2}}$,$2^{\frac{1}{2}}\phi^{-\frac{5}{2}}$), [ = ($2^{-1}\phi^{-1}$,$-2^{\frac{1}{2}}\phi^{-\frac{1}{2}}$,$2^{-1}5^{\frac{1}{4}}\phi^{-\frac{5}{2}}$), v = ($2^{-1}\phi^{-1}$,$-2^{\frac{1}{2}}\phi^{-\frac{1}{2}}$,$2^{-1}5^{\frac{1}{4}}\phi^{\frac{1}{2}}$), ! = ($2^{-1}\phi^{-1}$,$2^{\frac{1}{2}}\phi^{\frac{3}{2}}$,$2^{-1}5^{\frac{1}{4}}\phi^{\frac{1}{2}}$), ] = ($2^{-1}5^{\frac{1}{4}}\phi^{\frac{1}{2}}$,$2^{-1}\phi^{-1}$,$2^{\frac{1}{2}}\phi^{-\frac{1}{2}}$), _ = ($2^{-1}5^{\frac{1}{4}}\phi^{\frac{1}{2}}$,$2^{-1}\phi^{-1}$,$-2^{\frac{1}{2}}\phi^{\frac{3}{2}}$), % = ($2^{-1}5^{\frac{1}{4}}\phi^{\frac{1}{2}}$,$-2^{-1}\phi^{-1}$,$-2^{\frac{1}{2}}\phi^{-\frac{1}{2}}$), ’ = ($2^{-1}5^{\frac{1}{4}}\phi^{\frac{1}{2}}$,$-2^{-1}\phi^{-1}$,$2^{\frac{1}{2}}\phi^{\frac{3}{2}}$), g = ($2^{-1}5^{\frac{1}{4}}\phi^{\frac{1}{2}}$,$2^{\frac{1}{2}}\phi^{-\frac{1}{2}}$,$-2^{-1}\phi^{-1}$), h = ($2^{-1}5^{\frac{1}{4}}\phi^{\frac{1}{2}}$,$-2^{\frac{1}{2}}\phi^{\frac{3}{2}}$,$-2^{-1}\phi^{-1}$), ‘ = ($2^{-1}5^{\frac{1}{4}}\phi^{\frac{1}{2}}$,$-2^{\frac{1}{2}}\phi^{-\frac{1}{2}}$,$2^{-1}\phi^{-1}$), { = ($2^{-1}5^{\frac{1}{4}}\phi^{\frac{1}{2}}$,$2^{\frac{1}{2}}\phi^{\frac{3}{2}}$,$2^{-1}\phi^{-1}$), ∖ = (1,$2^{\frac{1}{2}}\phi^{-\frac{5}{2}}$,$5^{\frac{1}{4}}\phi^{-\frac{3}{2}}$), | = ($-2^{-1}\phi^{-1}$,$-2^{\frac{1}{2}}\phi^{-\frac{5}{2}}$,$2^{-1}5^{\frac{1}{4}}\phi^{-\frac{5}{2}}$), } = ($-2^{-1}\phi^{-1}$,$2^{-1}5^{\frac{1}{4}}\phi^{-\frac{5}{2}}$,$-2^{\frac{1}{2}}\phi^{-\frac{5}{2}}$), ∼ = ($-2^{-1}\phi^{-1}$,$2^{-1}5^{\frac{1}{4}}\phi^{-\frac{5}{2}}$,$2^{\frac{1}{2}}\phi^{-\frac{1}{2}}$), +1 = ($-2^{-1}\phi^{-1}$,$2^{-1}5^{\frac{1}{4}}\phi^{\frac{1}{2}}$,$2^{\frac{1}{2}}\phi^{-\frac{1}{2}}$), +2 = ($-2^{-1}\phi^{-1}$,$2^{-1}5^{\frac{1}{4}}\phi^{\frac{1}{2}}$,$-2^{\frac{1}{2}}\phi^{\frac{3}{2}}$), +3 = ($-2^{-1}\phi^{-1}$,$2^{\frac{1}{2}}\phi^{-\frac{1}{2}}$,$2^{-1}5^{\frac{1}{4}}\phi^{-\frac{5}{2}}$), +4 = ($-2^{-1}\phi^{-1}$,$2^{\frac{1}{2}}\phi^{-\frac{1}{2}}$,$2^{-1}5^{\frac{1}{4}}\phi^{\frac{1}{2}}$), +5 = ($-2^{-1}\phi^{-1}$,$-2^{\frac{1}{2}}\phi^{\frac{3}{2}}$,$2^{-1}5^{\frac{1}{4}}\phi^{\frac{1}{2}}$), O = (−1,0,$5^{\frac{1}{4}}\phi^{\frac{3}{2}}$), Q = (−1,0,$5^{\frac{1}{4}}\phi^{-\frac{3}{2}}$), C = (−1,$5^{\frac{1}{4}}\phi^{\frac{3}{2}}$,0), / = (−1,$5^{\frac{1}{4}}\phi^{\frac{3}{2}}$,$-2^{\frac{1}{2}}\phi^{\frac{3}{2}}$), @ = (−1,$5^{\frac{1}{4}}\phi^{\frac{3}{2}}$,$2^{\frac{1}{2}}\phi^{-\frac{1}{2}}$), k = ($5^{\frac{1}{4}}\phi^{-\frac{3}{2}}$,$-2^{\frac{1}{2}}\phi^{-\frac{5}{2}}$,−1), w = (−1,$-2^{\frac{1}{2}}\phi^{\frac{3}{2}}$,$5^{\frac{1}{4}}\phi^{\frac{3}{2}}$), $ = (−1,$2^{\frac{1}{2}}\phi^{-\frac{1}{2}}$,$5^{\frac{1}{4}}\phi^{\frac{3}{2}}$), " = (−1,$2^{\frac{1}{2}}\phi^{-\frac{1}{2}}$,$5^{\frac{1}{4}}\phi^{-\frac{3}{2}}$), y = ($5^{\frac{1}{4}}\phi^{-\frac{3}{2}}$,$2^{\frac{1}{2}}\phi^{-\frac{5}{2}}$,1), E = (−1,$5^{\frac{1}{4}}\phi^{-\frac{3}{2}}$,0), < = (−1,$5^{\frac{1}{4}}\phi^{-\frac{3}{2}}$,$2^{\frac{1}{2}}\phi^{-\frac{1}{2}}$), Y = ($5^{\frac{1}{4}}\phi^{-\frac{3}{2}}$,$2^{\frac{1}{2}}\phi^{-\frac{1}{2}}$,−1), * = (−1,$5^{\frac{1}{4}}\phi^{-\frac{3}{2}}$,$-2^{\frac{1}{2}}\phi^{-\frac{5}{2}}$), a = ($5^{\frac{1}{4}}\phi^{-\frac{3}{2}}$,$-2^{\frac{1}{2}}\phi^{-\frac{1}{2}}$,1), r = ($5^{\frac{1}{4}}\phi^{-\frac{3}{2}}$,−1,$2^{\frac{1}{2}}\phi^{-\frac{5}{2}}$), c = ($5^{\frac{1}{4}}\phi^{-\frac{3}{2}}$,−1,$-2^{\frac{1}{2}}\phi^{-\frac{1}{2}}$), B = ($5^{\frac{1}{4}}\phi^{-\frac{3}{2}}$,−1,0), o = ($5^{\frac{1}{4}}\phi^{-\frac{3}{2}}$,1,$-2^{\frac{1}{2}}\phi^{-\frac{5}{2}}$), e = ($5^{\frac{1}{4}}\phi^{-\frac{3}{2}}$,1,$2^{\frac{1}{2}}\phi^{-\frac{1}{2}}$), F = ($5^{\frac{1}{4}}\phi^{-\frac{3}{2}}$,1,0), N = ($5^{\frac{1}{4}}\phi^{-\frac{3}{2}}$,0,−1), R = ($5^{\frac{1}{4}}\phi^{-\frac{3}{2}}$,0,1), u = (−1,$-2^{\frac{1}{2}}\phi^{-\frac{5}{2}}$,$5^{\frac{1}{4}}\phi^{-\frac{3}{2}}$), H = ($-2^{\frac{1}{2}}\phi^{\frac{3}{2}}$,0,1), 5 = ($-2^{\frac{1}{2}}\phi^{\frac{3}{2}}$,1,0), b = ($5^{\frac{1}{4}}\phi^{\frac{3}{2}}$,$2^{\frac{1}{2}}\phi^{-\frac{1}{2}}$,−1), U = ($5^{\frac{1}{4}}\phi^{\frac{3}{2}}$,$-2^{\frac{1}{2}}\phi^{\frac{3}{2}}$,−1), Z = ($5^{\frac{1}{4}}\phi^{\frac{3}{2}}$,$-2^{\frac{1}{2}}\phi^{-\frac{1}{2}}$,1), V = ($5^{\frac{1}{4}}\phi^{\frac{3}{2}}$,$2^{\frac{1}{2}}\phi^{\frac{3}{2}}$,1), f = ($5^{\frac{1}{4}}\phi^{\frac{3}{2}}$,−1,$-2^{\frac{1}{2}}\phi^{-\frac{1}{2}}$), & = ($5^{\frac{1}{4}}\phi^{\frac{3}{2}}$,−1,$2^{\frac{1}{2}}\phi^{\frac{3}{2}}$), 9 = ($5^{\frac{1}{4}}\phi^{\frac{3}{2}}$,−1,0), d = ($5^{\frac{1}{4}}\phi^{\frac{3}{2}}$,1,$2^{\frac{1}{2}}\phi^{-\frac{1}{2}}$), ^ = ($5^{\frac{1}{4}}\phi^{\frac{3}{2}}$,1,$-2^{\frac{1}{2}}\phi^{\frac{3}{2}}$), D = ($5^{\frac{1}{4}}\phi^{\frac{3}{2}}$,1,0), L = ($5^{\frac{1}{4}}\phi^{\frac{3}{2}}$,0,−1), P = ($5^{\frac{1}{4}}\phi^{\frac{3}{2}}$,0,1), 7 = ($2^{\frac{1}{2}}\phi^{-\frac{1}{2}}$,1,0), J = ($2^{\frac{1}{2}}\phi^{-\frac{1}{2}}$,0,1).

### Appendix A.2. 4-dim MMPHs

**10-7**       1234,56,764,89A7,853,91,A2.

**24-13** LMNO,HIJK,EFGK,BCDG,9ADJ,78NO,5678,56AC,34BF,249I,1234,6EHM,146L. 1 = (0,1,−1,0), 2 = (1,0,0,1), 3 = (1,0,0,−1), 4 = (0,1,1,0), 5 = (0,1,0,0), 6 = (0,0,0,1), 7 = (1,0,*i*,0), 8 = (*i*,0,1,0), 9 = (1,−1,1,−1), A = (1,0,−1,0), B = (1,1,−1,1), C = (1,0,1,0), D = (0,1,0,−1), E = (1,1,0,0), F = (1,−1,1,1), G = (−1,1,1,1), H = (1,−1,0,0), I = (1,1,−1,−1), J = (1,1,1,1), K = (0,0,1,−1), L = (1,0,0,0), M = (0,0,1,0), N = (0,1,0,*i*), O = (0,*i*,0,1).

**25-15** EC1N,NOPQ,PQLM,MG5A,A98K,KJ67,6734,4HBE,ECDB,ED59,12KO,LMGF,MF4, 8234,KJH. 1 = (1,−1,1,1), 2 = (1,0,−1,0), 3 = (0,1,0,0), 4 = (0,0,0,1), 5 = (1,−1,1,−1), 6 = (*i*,0,1,0), 7 = (1,0,*i*,0), 8 = (1,0,1,0), 9 = (1,1,−1,−1), A = (1,−1,−1,1), B = (0,1,−1,0), C = (1,0,0,−1), D = (1,0,0,1), E = (0,1,1,0), F = (1,−1,0,0), G = (0,0,1,1), H = (1,0,0,0), J = (0,1,0,−1), K = (0,1,0,1), L = (0,0,1,−1), M = (1,1,0,0), N = (1,1,−1,1), O = (1,1,1,−1), P = (1,−1,*i*,*i*), Q = (−1,1,*i*,*i*).

**29-16** QRST,MNOP,IJKL,EFGH,BCDL,9ADP,8ACT,7BGH,69FH,58EH,347L,246P,145T, 12BK,139O,238S. 1 = (1,1,1,1), 2 = (1,1,−1,−1), 3 = (1,−1,1,−1), 4 = (1,−1,−1,1), 5 = (1,0,0,−1), 6 = (1,0,1,0), 7 = (0,0,1,1), 8 = (1,0,0,1), 9 = (1,0,−1,0), A = (1,1,1,−1), B = (0,0,1,−1), C = (−1,1,1,1), D = (1,−1,1,1), E = (0,0,1,0), F = (0,0,0,1), G = (1,0,0,0), H = (0,1,0,0), I = (0,0,2,−1), J = (0,0,1,2), K = (1,−1,0,0), L = (1,1,0,0), M = (1,0,2,0), N = (2,0,−1,0), O = (0,1,0,−1), P = (0,1,0,1), Q = (1,0,0,2), R = (2,0,0,−1), S = (0,1,1,0), T = (0,1,−1,0).

**30-16** 1234,4567,789A,ABCD,DEFG,GHI1,1JB9,GQ68,3KM5,EPNC,IJL5,COQH,4LPN, DOKM,FTUQ,JRS2. 1 = (0,0,0,1), 2 = (1,0,0,0), 3 = (0,1,−1,0), 4 = (0,1,1,0), 5 = (1,0,0,1), 6 = (1,1,−1,−1), 7 = (1,−1,1,−1), 8 = (1,−1,−1,1), 9 = (1,1,0,0), A = (0,0,1,1), B = (1,−1,0,0), C = (1,1,1,−1), D = (1,1,−1,1), E = (−1,1,1,1), F = (0,1,0,−1), G = (1,0,1,0), H = (1,0,−1,0), I = (0,1,0,0), J = (0,0,1,0), K = (−1,i,i,1), L = (1,0,0,−1), M = (1,i,i,−1), N = (1,−i,i,1), O = (1,−1,1,1), P = (1,i,−i,1), Q = (0,1,0,1), R = (0,1,0,i), S = (0,i,0,1), T = (1,0,i,0), U = (i,0,1,0).

#### New 4-dim MMPH Masters, Their Coordinatizations, and Their Distributions

**60-72** master from {0,±1,ϕ} or from $\left\{0,\pm \phi ,\frac{1}{\phi }\right\}$:

1234,1256,1278,129A,13BC,13DE,13FG,1HI4,1JK4,1LM4,23NO,23PQ,23RS,2TU4, 2VW4,2XY4,Za34,Za56,Za78,Za9A,Z5bc,Zde6,afg6,ah6i,a5jk,alm6,no34,no56,no78, no9A,pq34,pq56,pq78,pq9A,TUBC,TUDE,TUFG,TBmc,TCdj,UrCs,UBek,UClb,UCtu,VWBC, VWDE,VWFG,HINO,HIPQ,HIRS,HNmb,HOej,IvOw,INdk,IOlc,IOxy,JKNO,JKPQ,JKRS,fgbc, XYBC,XYDE,XYFG,LMNO,LMPQ,LMRS,hbci,rmcs,vmbw,lmbc,dejk,mbxy,mctu.

Coordinatization of **60-72** obtained from {0,±1,ϕ}:

1 = (0,0,0,1), 2 = (0,0,1,0), Z = (0,0,1,1), a = (0,0,1,−1), n = (0,0,1,ϕ), p = (0,0,−1,ϕ), q = (0,0,ϕ,1), o = (0,0,ϕ,−1), 3 = (0,1,0,0), T = (0,1,0,1), U = (0,1,0,−1), V = (0,1,0,ϕ), H = (0,1,1,0), I = (0,1,−1,0), J = (0,1,ϕ,0), f = (ϕ,ϕ,−1,−1), X = (0,−1,0,ϕ), L = (0,−1,ϕ,0), h = (ϕ,ϕ,1,1), Y = (0,ϕ,0,1), W = (0,ϕ,0,−1), M = (0,ϕ,1,0), r = (ϕ,−1,ϕ,−1), K = (0,ϕ,−1,0), v = (ϕ,−1,−1,ϕ), 4 = (1,0,0,0), N = (1,0,0,1), O = (1,0,0,−1), P = (1,0,0,ϕ), B = (1,0,1,0), C = (1,0,−1,0), D = (1,0,ϕ,0), 5 = (1,1,0,0), l = (1,1,1,1), d = (1,1,1,−1), e = (1,1,−1,1), m = (1,1,−1,−1), g = (1,1,ϕ,ϕ), 6 = (1,−1,0,0), j = (1,−1,1,1), b = (1,−1,1,−1), c = (1,−1,−1,1), k = (−1,1,1,1), 7 = (1,ϕ,0,0), s = (1,ϕ,1,ϕ), w = (1,ϕ,ϕ,1), 8 = (ϕ,−1,0,0), R = (−1,0,0,ϕ), F = (−1,0,ϕ,0), t = (ϕ,1,ϕ,1), i = (−1,−1,ϕ,ϕ), 9 = (−1,ϕ,0,0), u = (−1,ϕ,−1,ϕ), x = (ϕ,1,1,ϕ), y = (−1,ϕ,ϕ,−1), S = (ϕ,0,0,1), Q = (ϕ,0,0,−1), G = (ϕ,0,1,0), E = (ϕ,0,−1,0), A = (ϕ,1,0,0).

Coordinatization of **60-72** obtained from $\left\{0,\pm \phi ,\frac{1}{\phi }\right\}$:

1 = (0,0,0,ϕ), 2 = (0,0,ϕ,0), Z = (0,0,ϕ,ϕ), a = (0,0,ϕ,−ϕ), n = (0,0,ϕ,1/ϕ), p = (0,0,−ϕ,1/ϕ), q = (0,0,1/ϕ,ϕ), o = (0,0,1/ϕ,−ϕ), 3 = (0,ϕ,0,0), T = (0,ϕ,0,ϕ), U = (0,ϕ,0,−ϕ), V = (0,ϕ,0,1/ϕ), H = (0,ϕ,ϕ,0), I = (0,ϕ,−ϕ,0), J = (0,ϕ,1/ϕ,0), f = (1/ϕ,1/ϕ,−ϕ,−ϕ), X = (0,−ϕ,0,1/ϕ), L = (0,−ϕ,1/ϕ,0), h = (1/ϕ,1/ϕ,ϕ,ϕ), Y = (0,1/ϕ,0,ϕ), W = (0,1/ϕ,0,−ϕ), M = (0,1/ϕ,ϕ,0), r = (1/ϕ,−ϕ,1/ϕ,−ϕ), K = (0,1/ϕ,−ϕ,0), v = (1/ϕ,−ϕ,−ϕ,1/ϕ), 4 = (ϕ,0,0,0), N = (ϕ,0,0,ϕ), O = (ϕ,0,0,−ϕ), P = (ϕ,0,0,1/ϕ), B = (ϕ,0,ϕ,0), C = (ϕ,0,−ϕ,0), D = (ϕ,0,1/ϕ,0), 5 = (ϕ,ϕ,0,0), l = (ϕ,ϕ,ϕ,ϕ), d = (ϕ,ϕ,ϕ,−ϕ), e = (ϕ,ϕ,−ϕ,ϕ), m = (ϕ,ϕ,−ϕ,−ϕ), g = (ϕ,ϕ,1/ϕ,1/ϕ), 6 = (ϕ,−ϕ,0,0), j = (ϕ,−ϕ,ϕ,ϕ), b = (ϕ,−ϕ,ϕ,−ϕ), c = (ϕ,−ϕ,−ϕ,ϕ), k = (−ϕ,ϕ,ϕ,ϕ), 7 = (ϕ,1/ϕ,0,0), s = (ϕ,1/ϕ,ϕ,1/ϕ), w = (ϕ,1/ϕ,1/ϕ,ϕ), 8 = (1/ϕ,−ϕ,0,0), R = (−ϕ,0,0,1/ϕ), F = (−ϕ,0,1/ϕ,0), t = (1/ϕ,ϕ,1/ϕ,ϕ), i = (−ϕ,−ϕ,1/ϕ,1/ϕ), 9 = (−ϕ,1/ϕ,0,0), u = (−ϕ,1/ϕ,−ϕ,1/ϕ), x = (1/ϕ,ϕ,ϕ,1/ϕ), y = (−ϕ,1/ϕ,1/ϕ,−ϕ), S = (1/ϕ,0,0,ϕ), Q = (1/ϕ,0,0,−ϕ), G = (1/ϕ,0,ϕ,0), E = (1/ϕ,0,−ϕ,0), A = (1/ϕ,ϕ,0,0).

**86-152** master from {0,±1,i} or from {0,±i,1}:

1234,1256,1278,139A,13BC,1DE4,1FG4,23HI,23JK,2LM4,2NO4,PQ34,PQ56,PQ78, P5RS,PTU6,P6VW,P7XY,P7Za,Pbc8,P8de,Q5fg,Q5hi,Qjk6,Ql6m,Q7no,Q7pq,Qrs8, Qt8u,vw34,vw56,vw78,v5xy,v5z!,v"6#,v$6%,v7&’,v(8),w5*-,w5/:,w;6<,w=6>, w7?@,w[8∖,LM9A,LMBC,L9kS,LATf,LA@),LB:%,LBZu,L=zC,LCqe,M9Ug,M9(?,MAjR, MA]^,MB!>,MBpd,M$/C,MtCa,NO9A,NOBC,N9x<,N9cn,NA*#,NArY,NB&∖,NhCW,DEHI, DEJK,DHkR,DIUf,DI’∖,DJy<,DJau,D"*K,DKqd,EHTg,EH[&,EIjS,EI_‘,EJ-#,EJpe, E;xK,EtKZ,FGHI,FGJK,FH/%,FHbn,FIz>,FIrX,FJ?),FhKV,O9"-,O9sX,OA;y,OAbo, OBiV,O[C’,GH=!,GHsY,GI$:,GIco,GJiW,G(K@,jkRS,TUfg,TUhi,Tf(?,Tg’∖,;xy<, ;xau,;z!<,;cyn,Uf[&,Ug@),kR_‘,kS]^,"*-#,"*pe,"/:#,"r-Y,$*-%,$/:%,$/Zu, $b:n,=xy>,=z!>,=zpd,=r!X,lRSm,fgVW,*s#X,xb<o,zs>Y,/c%o,hiVW,rsXY,rsZa, bcno,bcpq,(?@),[&’∖,ty<Z,t:%a,tXYu,tZau,-#qd,!>qe,node,pqde.

Coordinatization of **86-152** obtained from {0,±1,i}:

1 = (0,0,0,1), 2 = (0,0,1,0), P = (0,0,1,1), Q = (0,0,1,−1), v = (0,0,1,i), w = (0,0,i,1), 3 = (0,1,0,0), L = (0,1,0,1), M = (0,1,0,−1), N = (0,1,0,i), D = (0,1,1,0), E = (0,1,−1,0), F = (0,1,i,0), O = (0,i,0,1), G = (0,i,1,0), 4 = (1,0,0,0), H = (1,0,0,1), I = (1,0,0,−1), J = (1,0,0,i), 9 = (1,0,1,0), A = (1,0,−1,0), B = (1,0,i,0), 5 = (1,1,0,0), j = (1,1,1,1), T = (1,1,1,−1), ; = (1,1,1,i), U = (1,1,−1,1), k = (1,1,−1,−1), " = (1,1,−1,i), $ = (1,1,i,1), = = (1,1,i,−1), l = (1,1,i,i), 6 = (1,−1,0,0), f = (1,−1,1,1), R = (1,−1,1,−1), * = (1,−1,1,i), S = (1,−1,−1,1), g = (−1,1,1,1), x = (1,−1,−1,i), z = (1,−1,i,1), / = (1,−1,i,−1), h = (1,−1,i,i), 7 = (1,i,0,0), r = (1,i,1,1), b = (1,i,1,−1), ] = (1,i,1,i), c = (1,i,−1,1), s = (1,i,−1,−1), ( = (1,i,−1,i), _ = (1,i,i,1), [ = (1,i,i,−1), t = (1,i,i,i), K = (i,0,0,1), C = (i,0,1,0), − = (−1,1,1,i), y = (−1,1,−1,i), ! = (−1,1,i,1), : = (−1,1,i,−1), i = (−1,1,i,i), < = (−1,−1,1,i), # = (i,i,i,1), % = (−1,−1,i,1), > = (i,i,1,i), m = (i,i,1,1), 8 = (i,1,0,0), n = (−1,i,1,1), X = (−1,i,1,−1), ? = (−1,i,1,i), Y = (−1,i,−1,1), o = (i,1,i,i), ^ = (i,1,i,1), & = (−1,i,i,1), ‘ = (i,1,1,i), p = (i,1,1,1), Z = (i,1,1,−1), a = (i,1,−1,1), q = (i,1,−1,−1), ’ = (i,1,−1,i), @ = (i,1,i,−1), u = (i,−1,1,1), d = (i,−1,1,−1), ∖ = (i,−1,1,i), e = (i,−1,−1,1), ) = (i,−1,i,1), V = (i,i,1,−1), W = (i,i,−1,1).

Distribution of critical KS MMPHs obtained (in an automated way on a supercomputer with the help of VECFIND, STATES01, MMPSHUFFLE, SHORTD, MMPTAG, and Linux shell and Emacs macros) from the **86-152** master, itself generated from {0,±1,i}:

**9** (number of hyperedges, *l*) 18 (number of vertices, *k*) (1) (number of MMPHs)
**11** 20 (2), 21 (2), 22 (4)
**13** 22 (2), 23 (6), 24 (33), 25 (40), 26 (35)
**15** 24 (1), 25 (3), 26 (52), 27 (208), 28 (573), 29 (542), 30 (265)
**16** 29 (1), 30 (7)
**17** 28 (4), 29 (103), 30 (860), 31 (2832), 32 (5011), 33 (3876), 34 (1325), 35 (1)
**18** 31 (7), 32 (11), 33 (25), 34 (15), 35 (19), 36 (5), 37 (2)
**19** 31 (2), 32 (112), 33 (724), 34 (2880), 35 (6701), 36 (9045), 37 (6139), 38 (1851), 39 (1)
**20** 34 (7), 35 (27), 36 (69), 37 (49), 38 (49), 39 (20), 40 (10), 41 (6)
**21** 34 (4), 35 (30), 36 (304), 37 (1318), 38 (3428), 39 (5807), 40 (6241), 41 (357), 42 (844), 43 (1)
**22** 37 (10), 38 (33), 39 (70), 40 (86), 41 (65), 42 (43), 43 (16), 44 (4), 45 (2)
**23** 38 (26), 39 (163), 40 (693), 41 (1617), 42 (3098), 43 (3749), 44 (3098), 45 (130), 46 (277), 47 (1)
**24** 39 (1), 40 (13), 41 (30), 42 (73), 43 (81), 44 (88), 45 (46), 46 (29), 47 (8), 48 (2), 49 (1)
**25** 40 (4), 41 (16), 42 (122), 43 (406), 44 (1005), 45 (1597), 46 (2127), 47 (175), 48 (1109), 49 (387), 50 (70), 51 (2)
**26** 41 (1), 42 (5), 43 (27), 44 (55), 45 (104), 46 (97), 47 (77), 48 (51), 49 (23), 50 (7), 52 (3)
**27** 42 (1), 43 (4), 44 (23), 45 (102), 46 (271), 47 (572), 48 (883), 49 (111), 50 (937), 51 (643), 52 (317), 53 (85), 54 (12)
**28** 44 (1), 45 (20), 46 (47), 47 (77), 48 (104), 49 (114), 50 (92), 51 (47), 52 (24), 53 (9), 54 (3), 55 (2) **29** 45 (1), 46 (9), 47 (40), 48 (118), 49 (230), 50 (349), 51 (506), 52 (53), 53 (462), 54 (314), 55 (149), 56 (83), 57 (18), 58 (2)
**30** 46 (1), 47 (10), 48 (33), 49 (79), 50 (139), 51 (157), 52 (141), 53 (97), 54 (44), 55 (20), 56 (7), 57 (1), 58 (3)
**31** 48 (2), 49 (35), 50 (86), 51 (157), 52 (250), 53 (272), 54 (297), 55 (26), 56 (206), 57 (148), 58 (69), 59 (35), 60 (13), 61 (1)
**32** 49 (4), 50 (22), 51 (75), 52 (149), 53 (193), 54 (196), 55 (126), 56 (69), 57 (37), 58 (17), 59 (12)
**33** 50 (5), 51 (24), 52 (67), 53 (146), 54 (226), 55 (219), 56 (196), 57 (15), 58 (126), 59 (85), 60 (59), 61 (25), 62 (10), 63 (1), 64 (2)
**34** 51 (4), 52 (18), 53 (54), 54 (130), 55 (220), 56 (200), 57 (188), 58 (12), 59 (64), 60 (31), 61 (9), 62 (2), 63 (1)
**35** 53 (8), 54 (41), 55 (118), 56 (184), 57 (240), 58 (219), 59 (158), 60 (11) 61 (67), 62 (33), 63 (18), 64 (7), 65 (1), 66 (4), 67 (1)
**36** 54 (5), 55 (26), 56 (91), 57 (194), 58 (232), 59 (226), 60 (166), 61 (89), 62 (39), 63 (23), 64 (4)
**37** 55 (1), 56 (23), 57 (71), 58 (155), 59 (226), 60 (248), 61 (185), 62 (10), 63 (58), 64 (22), 65 (15), 66 (7), 67 (3), 68 (1)
**38** 56 (2), 57 (14), 58 (48), 59 (143), 60 (194), 61 (221), 62 (192), 63 (10), 64 (62), 65 (22), 66 (5), 67 (1)
**39** 57 (3), 58 (10), 59 (35), 60 (84), 61 (174), 62 (210), 63 (156), 64 (13), 65 (60), 66 (29), 67 (8), 68 (5), 69 (2), 70 (2)
**40** 58 (2), 59 (2), 60 (18), 61 (55), 62 (140), 63 (167), 64 (139), 65 (10), 66 (64), 67 (20), 68 (6), 69 (11), 70 (1)
**41** 60 (2), 61 (7), 62 (34), 63 (89), 64 (125), 65 (116), 66 (101), 67 (67), 68 (34), 69 (13), 70 (3), 73 (1) **42** 62 (3), 63 (19), 64 (53), 65 (99), 66 (107), 67 (91), 68 (60), 69 (29), 70 (9), 72 (1)
**43** 63 (1), 64 (13), 65 (27), 66 (55), 67 (86), 68 (82), 69 (43), 70 (25), 71 (7), 72 (3), 73 (3), 75 (1)
**44** 64 (1), 65 (4), 66 (9), 67 (42), 68 (46), 69 (51), 70 (38), 71 (23), 72 (10), 73 (6), 75 (1)
**45** 66 (1), 67 (4), 68 (14), 69 (18), 70 (41), 71 (29), 72 (15), 73 (7), 74 (2)
**46** 66 (1), 68 (3), 69 (4), 70 (10), 71 (24), 72 (20), 73 (8), 74 (6), 76 (2)
**47** 69 (1), 70 (6), 71 (10), 72 (13), 73 (6), 74 (7), 75 (2), 76 (2)
**48** 71 (2), 72 (5), 73 (11), 74 (5), 75 (2), 76 (2), 77 (1), 78 (1), 79 (1)
**49** 74 (7), 75 (3), 76 (3)
**50** 73 (1), 76 (1), 77 (1), 81 (1)
**51** 75 (1)
**52** 76 (1)
111137 non-isomorphic critical KS MMPHs.

### Appendix A.3. 5-dim MMPHs

**29-16** 34125,56798,89CHG,GHF43,ABC95,DEF45,IJH94,KLG94,MNLB9,ONJA9,PMK79, POI69,QRIE4,SRKD4,TQJ24,TSL14 1 = (1,1,−1,1,0), 2 = (1,−1,1,1,0), 3 = (1,0,0,−1,0), 4 = (0,0,0,0,1), 5 = (0,1,1,0,0), 6 = (1,1,−1,0,1), 7 = (1,−1,1,0,1), 8 = (1,0,0,0,−1), 9 = (0,0,0,1,0), A = (1,−1,1,0,−1), B = (1,1,−1,0,−1), C = (1,0,0,0,1), D = (1,−1,1,−1,0), E = (1,1,−1,−1,0), F = (1,0,0,1,0), G = (0,0,1,0,0), H = (0,1,0,0,0), I = (1,0,1,0,0), J = (1,0,−1,0,0), K = (1,1,0,0,0), L = (1,−1,0,0,0), M = (0,0,1,0,−1), N = (1,1,1,0,1), O = (0,1,0,0,−1), P = (−1,1,1,0,1), Q = (0,1,0,1,0), R = (1,−1,−1,1,0), S = (0,0,1,1,0), T = (1,1,1,−1,0).

**11-7** 789AB,B65,513,32897,46A,134,245.

**21-7** (filled 11-7) 789AB,5CD6B,4EF6A,23789,1GH34,1IJ35,2KL45. 1 = (1,1,0,0,0), 2 = (0,1,0,0,0), 3 = (0,0,1,0,0), 4 = (0,0,0,1,1), 5 = (0,0,0,1,−1), 6 = (1,0,0,0,0), 7 = (1,0,0,−1,0), 8 = (0,0,0,0,1), 9 = (1,0,0,1,0), A = (0,1,−1,0,0), B = (0,1,1,0,0), C = (0,1,−1,1,1), D = (0,−1,1,1,1), E = (0,1,1,1,−1), F = (0,1,1,−1,1), G = (1,−1,0,1,−1), H = (1,−1,0,−1,1), I = (1,−1,0,1,1), J = (−1,1,0,1,1), K = (1,0,1,0,0), L = (1,0,−1,0,0).

### Appendix A.4. 6-dim MMPHs

**33-11** 123456,6789AB, BCD3EF, G5FMNO,8HIXWV, IAJD2K,KE4G7L,LH9JC1,MNOPQR, PQRSTU,STUVWX. 1=(0,0,1,1,ω,ω), 2 = (0,1,0,ω,1,ω), 3 = (0,1,ω,0,ω,1), 4 = (0,ω,1,ω,0,1), 5 = (1,0,0,0,0,0), 6 = (0,ω,ω,1,1,0), 7 = (1,1,ω,ω,0,0), 8 = (0,0,0,0,0,1), 9 = (ω,0,1,ω,1,0), A = (ω,1,0,1,ω,0), B = (1,ω,1,0,ω,0), C = (1,0,ω,0,1,ω), D = (ω,ω,0,0,1,1), E = (ω,1,1,0,0,ω), F = (0,0,0,1,0,0), G = (0,0,0,0,1,0), H = (0,1,0,0,0,0), I = (0,0,1,0,0,0), J = (1,0,0,ω,ω,1), K = (1,ω,0,1,0,ω), L = (ω,0,ω,1,0,1), M = (0,1,ω,0,0,ω), N = (0,ω,1,0,0,ω), O = (0,ω,ω,0,0,1), P = (1,0,0,ω,ω,0), Q = (ω,0,0,1,ω,0), R = (ω,0,0,ω,1,0), S = (0,1,1,0,0,ω), T = (0,1,ω,0,0,1), U = (0,ω,1,0,0,1), V = (1,0,0,1,ω,0), W = (1,0,0,ω,1,0), X = (ω,0,0,1,1,0)

**24-11** ($\bar{\mathrm{subhypergraph}}$ of 32-11) 123456,2568BM,MKAGCH,CGHJ9I,I7FNDO,NDOEL1, 3LDJCK,456789,ABCDEF,A5,A6.

**32-11** 123456,2568BM,MKAGCH,CGHJ9I,I7FNDO,NDOEL1,3LDJCK,456789,ABCDEF, AWVUT5,ASRQP6. 1 = (1,1,−1,0,0,1), 2 = (1,0,0,0,0,−1), 3 = (1,−1,1,0,0,1), 4 = (0,1,1,0,0,0), 5 = (0,0,0,0,1,0), 6 = (0,0,0,1,0,0), 7 = (1,−1,1,0,0,−1), 8 = (1,0,0,0,0,1), 9 = (1,1,−1,0,0,−1), A = (0,0,0,0,0,1), B = (0,0,1,0,0,0), C = (0,0,0,1,−1,0), D = (0,0,0,1,1,0), E = (1,−1,0,0,0,0), F = (1,1,0,0,0,0), G = (1,0,1,1,1,0), H = (1,0,1,−1,−1,0), I = (1,−1,−1,0,0,1), J = (0,1,0,0,0,1), K = (1,0,−1,0,0,0), L = (1,1,1,0,0,−1), M = (0,1,0,0,0,0), N = (0,0,1,1,−1,1), O = (0,0,1,−1,1,1), P = (0,1,0,0,1,0), Q = (1,0,1,0,0,0), R = (1,1,−1,0,−1,0), S = (1,−1,−1,0,1,0), T = (0,1,1,1,0,0), U = (1,0,1,−1,0,0), V = (1,1,−1,0,0,0), W = (1,−1,0,1,0,0).

### Appendix A.5. 8-dim MMPHs

**34-9** 12345678,9ABC5678,DEFG3478,HIJKLMFG,NOPQRMEC,STUQRLDB,VWUPJK9A, XYWTOI28,XYVSNH17. 1 = (0,0,1,1,1,1,0,0), 2 = (0,0,1,−1,1,−1,0,0), 3 = (0,0,0,1,0,−1,0,0), 4 = (0,0,1,0,−1,0,0,0), 5 = (0,1,0,0,0,0,0,0), 6 = (1,0,0,0,0,0,0,0), 7 = (0,0,0,0,0,0,0,1), 8 = (0,0,0,0,0,0,1,0), 9 = (0,0,0,1,0,0,0,0), A = (0,0,1,0,0,0,0,0), B = (0,0,0,0,0,1,0,0), C = (0,0,0,0,1,0,0,0), D = (1,−1,1,0,1,0,0,0), E = (1,1,0,1,0,1,0,0), F = (1,1,0,−1,0,−1,0,0), G = (−1,1,1,0,1,0,0,0), H = (0,1,−1,1,0,0,1,0), I = (1,0,1,1,0,0,0,−1), J = (1,0,0,0,1,1,0,1), K = (0,1,0,0,−1,1,−1,0), L = (0,0,1,0,−1,0,1,1), M = (0,0,0,1,0,−1,−1,1), N = (1,0,1,0,0,−1,1,0), O = (0,−1,1,0,0,1,0,1), P = (−1,1,0,0,0,0,1,1), Q = (1,0,−1,−1,0,0,0,1), R = (0,1,1,−1,0,0,−1,0), S = (1,0,0,1,−1,0,−1,0), T = (0,1,0,1,1,0,0,1), U = (1,1,0,0,0,0,1,−1), V = (0,1,0,0,1,−1,−1,0), W = (1,0,0,0,−1,−1,0,1), X = (1,1,0,−1,0,1,0,0), Y = (1,−1,−1,0,1,0,0,0).
